# Enhancing Cancer Treatment Through Combined Approaches: Photodynamic Therapy in Concert with Other Modalities

**DOI:** 10.3390/pharmaceutics16111420

**Published:** 2024-11-06

**Authors:** Gyeong Hong, Ji-Eun Chang

**Affiliations:** College of Pharmacy, Dongduk Women’s University, Seoul 02748, Republic of Korea

**Keywords:** combination cancer treatment, photodynamic therapy, lung cancer, breast cancer, cholangiocarcinoma, cervical cancer

## Abstract

This review explores the role of photodynamic therapy (PDT) as an adjunctive treatment for cancers, with a focus on its potential to enhance the effects of established therapies like chemotherapy, surgery, and radiotherapy. Given the limitations of conventional cancer treatments, PDT’s ability to improve therapeutic outcomes through combination strategies is examined. In cancers such as lung, breast, cholangiocarcinoma, and cervical, PDT shows promise in enhancing response rates, reducing recurrence, and minimizing adverse effects when used alongside standard modalities. This study highlights current findings on PDT’s mechanisms in complementing chemotherapy, augmenting surgical precision, and enhancing radiotherapeutic effects, thus offering a multi-faceted approach to cancer treatment. Additionally, insights into the clinical application of PDT in these cancers emphasize its potential for reducing tumor resistance and supporting more effective, personalized care. By providing an overview of PDT’s synergistic applications across diverse cancer types, this review underscores its emerging significance in oncology as a tool to address traditional treatment limitations. Ultimately, this review aims to inform and inspire researchers and clinicians seeking to refine and innovate cancer therapy strategies through PDT integration, contributing to the advancement of more effective, synergistic cancer treatments.

## 1. Introduction

Cancer continues to be a significant global health challenge [1], necessitating the relentless pursuit of effective treatment strategies across diverse cancer types. Despite significant advancements in the understanding of cancer biology and the development of various therapeutic strategies, the complex and heterogenous nature of this disease poses continuous challenges [2].

Traditional cancer treatments, including surgery, radiotherapy, and chemotherapy, have played crucial roles in extending patient survival and improving outcomes [3]. However, their limitations in terms of selectivity, invasiveness, and resistance have driven researchers to explore innovative approaches.

A multidisciplinary approach has emerged as a prominent strategy, combining various therapeutic modalities to address the multifaceted nature of cancer. Among these approaches, “photodynamic therapy (PDT)” has rapidly gained attention as a promising contender in the field of cancer therapy, particularly due to its potential synergistic interactions with conventional treatments [4].

**Figure 1:**
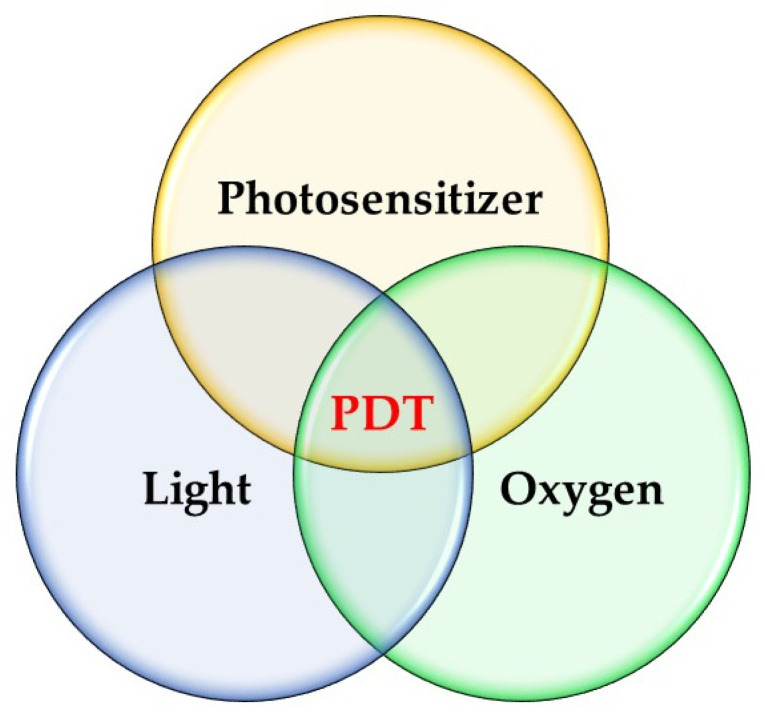
Key components of photodynamic therapy.

**Figure 2:**
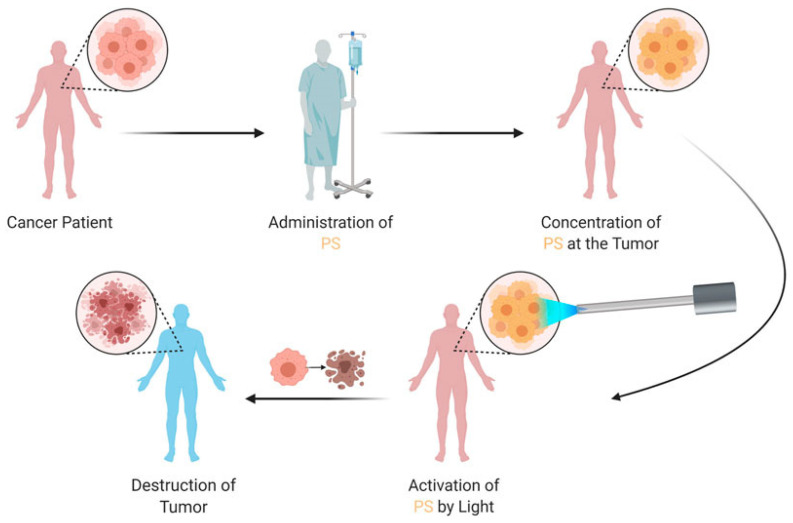
Application of photodynamic therapy in cancer patients (PS: photosensitizer) [6].

PDT comprises three essential components: a photosensitizer, light, and oxygen (Figure 1). Photosensitizers act as catalysts, converting molecular oxygen into reactive oxygen species such as singlet oxygen, hydroxyl radicals, and superoxide anions upon light absorption. PDT has garnered significant interest due to its unique mechanism of action, which selectively targets cancer cells while sparing healthy tissue. This selectivity is primarily due to the photosensitizing agents used in PDT, which accumulate preferentially in tumor cells. When activated by light, these agents produce reactive oxygen species, especially singlet oxygen, causing localized cell death [5]. This makes PDT a minimally invasive treatment option with fewer systemic side effects compared to traditional methods like chemotherapy or radiation. Figure 2 represents how PDT applies to cancer patients [6].

The growing body of evidence suggests that PDT not only serves as a standalone therapy but also enhances the efficacy of conventional cancer treatments through its synergistic potential. One of the most promising aspects of PDT is its ability to enhance the effects of other therapeutic modalities. When combined with chemotherapy or radiotherapy, PDT can reduce the required doses of both treatments, leading to fewer side effects and decreased drug resistance. Additionally, enhanced overall treatment efficacy can be achieved [7]. In combination with surgery, PDT can serve as a preoperative or intraoperative treatment. Preoperative PDT reduces tumor size, improving surgical outcomes [8], while intraoperative PDT helps lower the risk of cancer recurrence after surgery [9].

**Figure 3:**
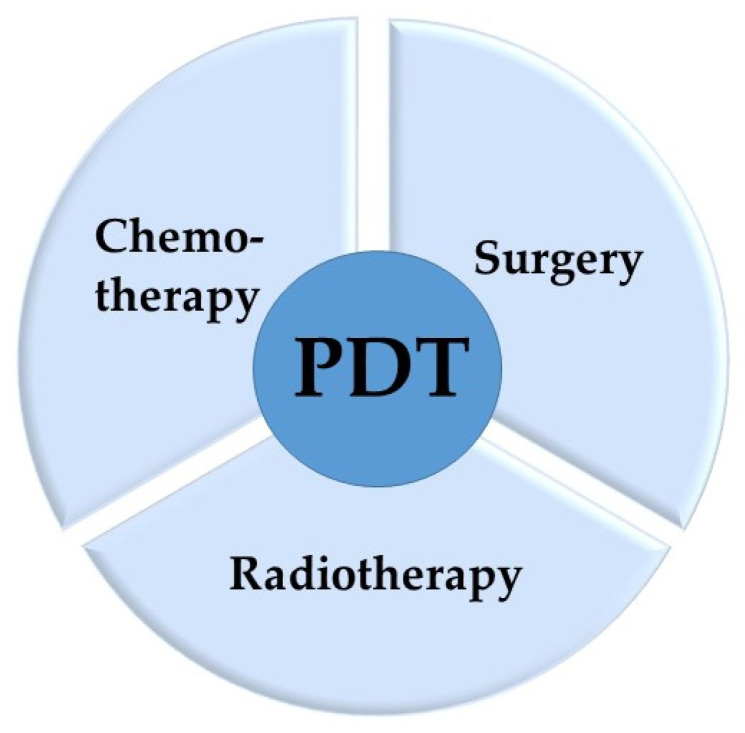
Combination approaches involving PDT and conventional cancer treatments.

This review aims to present and analyze the outcomes of combination therapy involving PDT and traditional approaches such as chemotherapy, surgery, and radiotherapy (Figure 3). Four representative cancer types—lung cancer, breast cancer, cholangiocarcinoma, and cervical cancer—are highlighted, selected for their active and advancing research in PDT treatments, particularly in cases where PDT-based combination therapies are frequently reported. By focusing on these cancers, this review explores the interplay and potential synergies between PDT and conventional therapeutic methods.

The contemporary cancer therapy landscape is marked by an intricate interplay between multiple therapeutic approaches aiming to provide more effective responses against cancer cells. The investigation at hand seeks to unravel the interactions between PDT and various other treatments, thereby contributing novel insights to the realm of cancer therapy.

As we navigate the intricate landscape of cancer treatment, the convergence of PDT with traditional approaches offers a promising avenue for overcoming existing challenges and improving patient outcomes. By dissecting the nuances of these combinations within the context of specific cancer types, this paper endeavors to contribute to the ongoing evolution of effective and tailored cancer therapies.

## 2. PDT in Lung Cancer and Combination Therapies

Lung cancer is classified into two broad groups, which grow and metastasize differently, small cell lung carcinoma (SCLC) and non-small cell lung carcinoma (NSCLC), which are distinguished based on the appearance of the cells when viewed under a microscope. NSCLC is the most common type of lung cancer, accounting for approximately 85% of all cases [10]. Treatment options include surgery, chemotherapy, and radiation therapy, which are determined according to the subtype [3].

PDT is an approved therapy option for symptom relief in patients with early-stage lung cancer (stage 0 and stage 1), tracheal cancer, and complete or partially obstructive endobronchial lung cancer in Japan, the United States, and many other countries. In the 1980s, it was applied for the first time in lung cancer patients after undergoing basic research in Japan. Two years later, in 1982, Hayata and colleagues reported the results of PDT treatment in 16 patients with lung cancer [11]. The US Food and Drug Administration (FDA) approved PDT for the treatment of microinvasive endobronchial NSCLC in early 1998 and for advanced partially obstructing endobronchial lung cancer in late 1998. There has been a growing interest in using PDT either alone or in combination with standard therapeutic or palliative modalities for the treatment of NSCLC [12].

PDT is mainly used for patients with early-stage NSCLC. It can also be used to treat obstructing endobronchial lesions or reduce the extent of surgery needed for patients with advanced-stage NSCLC [13]. However, PDT may not be suitable for patients with SCLC due to their tendency for distant metastases. Nevertheless, PDT can be considered for palliative care for symptomatic SCLC patients who have a progressive disease despite standard treatments such as chemotherapy [14]. Table 1 summarizes the combined therapies with PDT in lung cancer.

### 2.1. PDT with Chemotherapy in Lung Cancer

Chemotherapy is a frequently utilized treatment modality for a variety of cancers, and its effectiveness has greatly improved in recent years. However, it is associated with severe side effects due to reduced selectivity for cancerous cells, and its therapeutic efficiency is hindered by chemo-resistance, which can lower its effectiveness in certain types of cancers [15].

Cacaccio et al. demonstrated that combination PDT with doxorubicin (DOX) is superior to monotherapy. This report discussed the treatment of lung cancer, both with and without DOX chemotherapy, across various lung tumors and at different doses in both in vivo and in vitro settings. For their in vitro studies, they tested varying doses of photosensitizer 1, [(methyl-3-(1-meta-iodo-benzyloxy) ethyl-3-devinylpyropheophorbide-a], and DOX on lung cancer cells (A549 and H460). To evaluate the synergistic effects of photosensitizer 1 and DOX, A549 cells were treated with different drug doses. According to the in vitro model, the most effective ratio for DOX and photosensitizer is 2:1 in terms of efficacy. H460 cells exhibited a similar response to A549 cells, except that the regions of antagonism were more widespread. Cacaccio’s group also demonstrated which delivery vehicle for the photosensitizer had more impact on the mode of action of PDT in combination with DOX therapy via a synergistic study conducted using both the Tween and Pluronic (1% Tween 80/5% dextrose and 2% Pluronic F-127 in PBS) formulations of photosensitizer 1. The synergistic effects of photosensitizer 1 and DOX were similar when photosensitizer 1 was dissolved in Tween with the Pluronic formulation. However, photosensitizer 1 demonstrated high tumor specificity and stability when formulated with Pluronic at a concentration of 2%.

They also performed an in vivo study to determine the optimal time for light irradiation to tumors and establish the treatment parameters of PDT. Whole-body fluorescence imaging of the desired photosensitizer was performed at various time points in four PDX models (NSCLC 148070, NSCLC 0229042, SCC 14541, and lung adenocarcinoma 15021). To evaluate the potential synergistic effects of PDT and chemotherapy, they first conducted PDT on female SCID mice bearing SCLC 14541 tumors. Photosensitizer 1 was administered, followed by light exposure 24 h later. Subsequently, the mice were treated with a single intravenous injection of DOX. The mice were monitored for 60 days following PDT treatment. The combination of photosensitizer 1-PDT and a single dose of DOX showed promising results in increasing long-term treatment in SCID mice with SCLC 14541 tumors. These findings suggest that optimizing treatment parameters, including variable doses of photosensitizer and either DOX or cisplatin, could further enhance long-term cure rates while reducing toxicity in future studies [16].

Chan et al. reported that a low dose of cisplatin in combination with PDT can be an effective treatment modality in the therapy of SCLC patients [14]. Cisplatin has been commonly used in treatment of limited-stage SCLC patients as well as in the first-line chemotherapy for extensive-stage SCLC patients [17]. SCLC is an aggressive type of lung cancer that presents an overall 5-year survival rate below 10%. Even though chemotherapy using cisplatin has been shown to be effective in SCLC treatment, the usual dosage of cisplatin causes adverse side effects such as severe neurotoxicity, nephrotoxicity, and adverse gastrointestinal effects. Thus, the study investigated whether the combination of low-dose cisplatin and PDT could effectively reduce cell viability and cell migration through their synergic induction of reactive oxygen species (ROS) in SCLC cells. In the combined treatment group, EtNBS-COOH was injected into the tumor 24 h later, and the mice were then subjected to PDT. Cisplatin and PDT had synergistic inhibitory effects on NCI-H446 cell viability and migration. Both cisplatin and PDT induced an increase in ROS production in cultured NCI-H446 cells. Notably, co-treatment with cisplatin and PDT resulted in a more significant increase in intracellular ROS levels. This treatment approach has the potential to cause fewer side effects compared to traditional chemotherapy using cisplatin at a relatively high dose. These in vitro findings may provide valuable guidance for the potential combined use of cisplatin and PDT as a therapy for treating SCLC in human patients [14].

Akopov et al. designed the study as a prospective randomized trial to compare the efficacy of preoperative endobronchial PDT combined with chemotherapy versus preoperative chemotherapy alone for downgrading IIIA and IIIB NSCLC (main bronchus/distal trachea involvement). The chemotherapy treatment plan involved administering paclitaxel at a dose of 200 mg/m^2^ intravenously over 3 h and carboplatin at a dose of AUC < 6 intravenously over 1 h every 21 days for a total of three cycles. The dosage of each chemotherapy drug was adjusted based on the observed levels of toxicity. Patients received Radachlorin^®^ at a dose of 1 mg/kg. This was followed by endoluminal irradiation of the tumor using a 662 nm laser light. The response to neoadjuvant treatment was evaluated two weeks after completion using the same diagnostic techniques, except for mediastinoscopy. Patients who showed a positive response (complete or partial) to the neoadjuvant therapy were offered surgical treatment within three to six weeks after the final cycle. The completeness of resection was significantly greater in the PDT group compared to the group that did not receive PDT. This study showed that neoadjuvant PDT in combination with chemotherapy is safe and effective. It also enabled conversion of patients into surgery candidates and improved the completeness of resection in those with stage III central NSCLC [8].

### 2.2. PDT with Surgery in Lung Cancer

Over the past two decades, preoperative chemotherapy has become more common in order to reduce the size of tumors and increase the likelihood of successful surgery. Although there is currently no strong evidence to suggest that preoperative chemotherapy can completely transform a tumor that was initially considered inoperable into one that can be surgically removed, multiple clinical trials of neoadjuvant chemotherapy since the 1990s have shown a slight improvement in survival rates compared to using surgical methods alone [18,19,20,21]. PDT is used in conjunction with surgery in two ways: as a preoperative treatment and as an intraoperative treatment.

Preoperative PDT is a promising option to help reduce the size of tumors before surgery and increase the chances of successful surgical removal. Several studies have shown that preoperative PDT can result in less extensive pulmonary resections [8]. Kato et al. studied fifteen lung cancer patients who underwent combined preoperative PDT and surgery. PDT was performed prior to the surgery with the aim of either reducing the extent of resection required or improving the operability of the tumor. The initial objective of PDT was successfully achieved in 11 out of the 15 patients who received the treatment. In 4 out of the 5 patients who were initially deemed inoperable, PDT made it possible to convert their condition to be operable. Among the 10 patients who were originally candidates for pneumonectomy, PDT made it possible to reduce the extent of resection needed to lobectomy or bilobectomy in 7 cases. These findings suggest that PDT could be an important complementary treatment modality for advanced lung cancers when used in conjunction with surgery and other therapies [22].

Okunaka’s group also investigated the use of preoperative PDT to either reduce the extent of resection required or improve operability in a cohort of 26 patients. Overall, in 22 out of 26 patients treated with PDT, the initial purpose of either reducing the extent of resection or converting an inoperable disease to an operable status was achieved. Of the five patients with an originally inoperable disease, four were converted to an operable condition by PDT. While 21 patients were originally candidates for pneumonectomy, it was possible to reduce the extent of resection to lobectomy or sleeve lobectomy in 18 cases. Nonetheless, three patients subsequently died as a result of distant metastasis, and recurrence was recognized in two other cases. By utilizing preoperative PDT as a form of laser irradiation, the survivability of T3 cases with main bronchus invasion treated solely by surgery has shown a significant increase from 50.9% to 60.0%. This notable improvement suggests that PDT could be a valuable option for the management of advanced lung malignancies [23].

Allison et al. reported a case study about a patient who had peripheral NSCLC. According to the protocol, the lesion had to be located at a distance of more than 2 cm from any major vessel, structure, or visceral pleura and be suitable for surgical resection. As planned, surgical resection via lobectomy was performed on post-PDT day 30. The patient recovered well after undergoing surgery and PDT and did not experience any long-term adverse effects related to either treatment. At the 90-day follow-up, the patient remained free of any signs of disease on both imaging and physical examination, thus concluding the study [24]. PDT can be used as a definitive treatment for peripheral tumors, or as demonstrated in this case, in combination with surgery using minimally invasive techniques such as wedge resection. PDT has also been found to be highly effective when used in combination with high-dose radiation therapy in both the upper and lower lobes of the lung, resulting in a 97% complete response rate [25]. Therefore, Allison’s group recommended that further evaluation of the combination of PDT and radiotherapy be conducted for peripheral lung tumors. Moreover, with the advent of numerous new bronchoscopy technologies, the combined use of these technologies with PDT should also be further explored [24].

Intraoperative PDT represents a potential solution for reducing the incidence of recurrence or microscopic residual disease following surgical resections [9,26]. Several studies have reported that PDT during the surgery may have the potential to enhance the survival rates of patients with malignant pleural mesothelioma. Friedberg et al. evaluated the effectiveness and toxicity of pleural PDT in patients with NSCLC metastatic to the pleura. In this phase II study, a total of 22 patients were enrolled and treated with the same PDT regimen. Pleural PDT was administered to 20 patients, while two of them did not receive it. The median follow-up period for all 22 patients was 33.6 months. Overall survival and progression-free survival were calculated for all 22 patients, as well as for the 20 patients who underwent PDT. The median overall survival was 21.7 months, and the 1-year survival rate was 68%. The median progression-free survival was 6.6 months, and the 1-year progression-free survival rate was 28% [27]. Patients with NSCLC and pleural spread who have a good performance status are known to have a survival range of 6 to 9 months [28,29,30]. This is in contrast to other stage III patients with locally advanced NSCLC, who are typically treated with chemoradiotherapy and have a median survival of 15 to 18 months [31]. This study utilized intraoperative pleural PDT as a component of a multimodal treatment approach for patients with NSCLC and pleural carcinomatosis. The findings from the analysis suggest that this approach shows promise. The authors concluded that, by carefully administering the appropriate light dosage, it is feasible to perform intraoperative hemithoracic PDT with an acceptable level of morbidity [27].

Chen et al. researched the clinical features and treatment results of patients diagnosed with either lung cancer or thymoma and pleural seeding who underwent pleural PDT and surgery between 2005 and 2013 at the University of Pennsylvania (phase II trial). A total of 18 patients were included in the study. Among them, 10 patients had pleural dissemination originating from lung cancer, while 8 patients had thymoma as the source of their pleural dissemination. The PDT group was administered the therapy by moving the device around the hemithorax. The median overall survival for the lung cancer patients in the PDT group was 39.0 months. During the same study period, a control group of NSCLC patients (*n* = 51) with pleural spread underwent traditional chemotherapy, radiotherapy, or targeted therapy [32]. The mean survival time for the non-PDT patients in the control group was 17.6 months. With appropriate patient selection, it appears feasible to combine radical surgical resection with intrapleural PDT for patients with NSCLC or thymoma with pleural spread. This approach has the potential to provide a survival benefit [32].

Jung and Kim et al. reported a case of a 70-year-old male patient who was diagnosed with squamous cell carcinoma in the right upper lung and an endobronchial lesion that extended into the distal trachea. In light of the patient’s preoperative examination results, which showed distal tracheal invasion and a high risk of morbidity or mortality after carinal resection and reconstruction, the medical team decided to perform intraoperative PDT following gross total resection of the tumor. The patient received an intravenous injection of Photofrin^®^ 48 h prior to the surgery. During the intraoperative PDT procedure, the affected area was irradiated. As per the decision of the multidisciplinary team, the patient underwent four cycles of adjuvant chemotherapy using a regimen of high-dose methotrexate, cisplatin, and DOX. Thirteen months following the surgery, the patient was still alive, and there was no evidence of cancer recurrence [33].

### 2.3. PDT with Radiotherapy in Lung Cancer

Radiotherapy is a crucial form of treatment for lung cancer that can be used in all stages of the disease and for patients with varying levels of performance status [34]. In fact, modelling suggests that 77% of all lung cancer patients will require radiotherapy at some point during their cancer journey based on evidence-based indications [35]. PDT is typically used as an endobronchial treatment for patients with early-stage NSCLC. Its main purpose is to definitively treat endobronchial, radiographically occult, or synchronous primary carcinomas. Combining PDT with external beam radiation therapy or endobronchial brachytherapy can greatly enhance symptomatic relief and extend the duration of local control for patients with symptomatic obstructing NSCLC. While the most effective sequence for combined therapy is not yet established, administering high-dose-rate (HDR) brachytherapy before PDT and delivering PDT before external beam radiation therapy may result in the best local control [13]. Numerous studies have been conducted on the use of PDT in combination with radiotherapy for the treatment of lung cancer.

Weinberg et al. reviewed the effectiveness of combining PDT and HDR in treating patients with symptomatic obstruction caused by endobronchial NSCLC. Weinberg’s team identified nine patients who underwent combined PDT and HDR for endobronchial cancers and reviewed their medical records [36]. Research has indicated that utilizing multiple bronchoscopic modalities is more effective than relying on a single modality, and sequential PDT/HDR treatments have been shown to be effective in controlling the growth of limited endobronchial carcinoma [25,37]. Researchers proposed that the sequential use of HDR and PDT could improve local tumor control and reduce the overall number of interventions required to treat endobronchial NSCLC. In this study, the authors reported on the outcomes of intentionally combining these two treatments in a group of nine patients. The study involved two treatment groups: TG-1 (*n* = 7), in which patients received HDR before PDT, and TG-2 (*n* = 2), in which patients received PDT before HDR. Combining HDR and PDT procedures can result in more prolonged local tumor control, although it may not be the optimal approach for emergent situations. The aim of this approach is to maximize the duration of tumor control while minimizing the number of interventions required for the patient. In addition, the synergistic interaction between these two treatments can be explored. When considering the preferred order of these treatments, it is argued that using HDR first may have practical and theoretical advantages. HDR can be performed immediately upon diagnosis of endobronchial disease, while a 48-h delay is required for photosensitizer accumulation in PDT [36]. Moreover, HDR’s ability to control active bleeding may justify its use before PDT [38]. Additionally, prior HDR treatment can shrink the tumor and make its surfaces more clearly visible, thereby assisting in the accurate placement of PDT. However, it should be noted that HDR can reduce the tumor’s vascularization over time, which could inhibit photosensitizer uptake and decrease the effectiveness of subsequent PDT treatments [25]. To circumvent this theoretical effect, closer spacing of the treatments in time may be necessary. When HDR was performed before PDT in TG-1, all but one patient had prolonged clinical and pathological local tumor control for 3 months to 5+ years, with only occasional surveillance bronchoscopy needed for biopsy samples and reverse bronchial contraction. None of these patients experienced hemoptysis. It is worth noting that the patient who failed treatment in TG-1 already had systemic (stage 4) disease. In contrast, the patients in TG-2 had less satisfactory outcomes, with successful local palliation in only one patient lasting for 10 weeks. This provides evidence that sequential HDR and PDT using bronchoscopic treatment can offer prolonged disease control in patients with endobronchial NSCLC, during which little or no endobronchial intervention is needed. The study also suggests that treatment order and spacing may be crucial variables in determining the efficacy of this approach. Specifically, using HDR before PDT and delivering the treatments no more than a month apart yielded more satisfactory results in this patient population, although it is impossible to know which effect played a larger role in this study [36].

Wang et al. introduced X-ray-induced PDT (X-PDT), which utilizes X-rays as an energy source to activate a PDT process [39]. Chen et al. first proposed the idea of using X-rays to address the limited tissue penetration of PDT in 2006 [40]. The enhanced cell-killing effectiveness of X-PDT compared to radiotherapy is attributed to synergy, which is especially evident in H1299 cells, a type of radioresistant NSCLC cell. Experiments have demonstrated this enhanced efficacy not only in vitro but also in vivo, with a subcutaneous tumor model and when H1299 cells were percutaneously implanted into the lung. These findings provide valuable insights into the potential of X-PDT as a novel treatment modality and its future translation into clinical practice [39]. In their earlier study, the researchers utilized their own developed drug, a co-loaded silica nanoparticle mesoporous formulation (MC540-SAO:Eu@mSiO_2_), which combined SrAl2O4:Eu2+ nanoparticles (SAO:Eu), a scintillator that converts X-ray photons to visible photons, with MC540, a photosensitizer that matches the excitation wavelength [41]. This formulation was capable of producing singlet oxygen when exposed to X-ray radiation, leading to effective cancer cell death even in the presence of thick tissues. Wang’s team initially examined the effects of X-PDT on radioresistant NSCLC H1299 cells. In their experiments, they observed that radiotherapy alone did not cause substantial cell death within 24 h. However, when the cells were exposed to X-ray irradiation following incubation with MC540-SAO:Eu@mSiO_2_, a significant decrease in cell viability was observed, indicating the effectiveness of X-PDT. The impact of X-PDT on DNA and lipid membranes was also investigated. When cells were treated with radiotherapy alone, a moderate level of apoptosis was observed after 24 h, but no necrosis was detected. In contrast, when cells were exposed to MC540-SAO:Eu@mSiO_2_ along with radiation, extensive cell necrosis was observed. X-PDT combines the effects of radiotherapy and PDT, which target different cellular compartments, namely DNA and unsaturated membrane lipids, respectively. This combination treatment is believed to result in a synergistic effect, leading to more efficient cancer cell killing. The impact of tissue thickness on the efficacy of X-PDT was investigated in vivo using mouse subcutaneous tumor models established with H1299 cells. These findings suggest that tissue thickness can still impact the efficacy of X-PDT, although to a lesser extent than with PDT, which loses its efficacy beyond 1 cm thickness [39]. This impact can be compensated for by increasing radiation doses and is less of a concern in the clinical setting, where deep-penetrating megavoltage X-rays are typically used [42]. As a result, X-PDT improved efficacy in tumor therapy, particularly for radioresistant cells. X-PDT not only reduces the short-term viability of cancer cells but also their clonogenicity in the long-term, making it a promising radiosensitizing method. Additionally, X-PDT can be used to suppress tumors located deep within tissues. These findings and advances have great value in the development of X-PDT as a novel treatment method for cancer [39].

**Table 1:** Combined therapies with PDT in lung cancer.

Combined Therapy		Photosensitizer	Dose	Light Source	Study Type	Ref.
Chemotherapy	doxorubicin (2.5 mg/kg)	[(methyl-3-(1-meta-iodo-benzyloxy) ethyl-3-devinylpyropheophorbide-a]	1 μM/kg	665 nm (135 J/cm^2^)	in vitro, in vivo	[16]
	cisplatin (5 mg/kg)	EtNBS-COOH	2 μM in 200 μL	630–650 nm (15 J/cm^2^)	in vivo	[14]
	paclitaxel (200 mg/m^2^), carboplatin (AUC < 6)	Radachlorin^®^	1 mg/kg	662 nm (150 J/cm^2^)	clinical	[8]
Surgery	preoperative PDT	HpD	2.5–3.0 mg/kg	630 nm (60–600 J/cm^2^)	clinical	[22]
		Photofrin^®^	2.0 mg/kg	630 nm (100–800 J/cm^2^)	clinical	[23]
		Photofrin^®^	2 mg/kg	630 nm (200 J/cm^2^)	clinical	[24]
	intraoperative PDT	porfimer sodium	2 mg/kg	630 nm (30 J/cm^2^)	clinical	[27]
		Photofrin^®^	-	630 nm (30 J/cm^2^)	clinical	[32]
		Photofrin^®^	2 mg/kg	630 nm (120 J/cm^2^)	clinical	[33]
Radiotherapy	iridium-192 (5 Gy)	Photofrin^®^	2 mg/kg	630 nm (200 J/cm^2^)	clinical	[36]
	X-ray (5 Gy)	MC540-SAO:Eu@mSiO_2_	50 µg/mL, 4.25 mg/kg		in vitro, in vivo	[39]

## 3. PDT in Breast Cancer and Combination Therapies

Breast cancer is the most prevalent cancer in women, and it is a significant cause of cancer-related deaths in this population. Surgery is the primary approach for tumor removal in the majority of women diagnosed with breast cancer, with options including breast-conserving surgery or mastectomy (complete breast removal). Following surgery, adjuvant treatments such as radiotherapy or chemotherapy are often administered. In recent years, researchers have shown greater interest in combining multiple anti-tumor therapies to improve patient outcomes and reduce unwanted side effects. With recent technological advancements in PDT, its applications in breast cancer patients have expanded. Moreover, the breast area is easily accessible for PDT. However, it is crucial to use the appropriate energy levels in clinical studies, as the optimal energy dosage can vary for each tissue and organ. In a study conducted by Ahn et al., it was found that PDT with an energy dosage of 90 J/cm^2^ was effective in breast cancer models that were less than 10 mm in size [43]. While PDT has shown potential in curing early-stage tumors, its localized effect limits its ability to achieve a cure for advanced metastatic cancers. Therefore, combining PDT with chemotherapy is one of several strategies proposed to enhance the therapeutic outcome of low-dose chemotherapy, minimize side effects, and reduce the likelihood of developing drug resistance [44,45]. Table 2 summarizes the combined therapies with PDT in breast cancer.

### 3.1. PDT with Chemotherapy in Breast Cancer

Chemotherapy is a cancer treatment that utilizes cancer-killing drugs delivered through intravenous infusion or orally. These drugs travel through the bloodstream to reach cancer cells in various parts of the body. Chemotherapy may be recommended in several situations for breast cancer patients, such as after surgery (adjuvant chemotherapy), before surgery (neoadjuvant chemotherapy), or for advanced breast cancer. Combinations of multiple drugs are usually used for the most effective treatment, especially for adjuvant and neoadjuvant therapy. Although several combinations are currently in use, there is no clear evidence indicating any particular combination as more effective. Common chemotherapeutic agents for early breast cancer include taxanes (such as docetaxel and paclitaxel) and anthracyclines (epirubicin and DOX), which may be combined with other drugs like cyclophosphamide and fluorouracil. Platinum agents like cisplatin and carboplatin have also shown efficacy in treating breast cancer. Recent studies have explored the combination of PDT and traditional chemotherapy for treating breast cancer [46].

In vitro studies have shown that low doses of cisplatin, which are unlikely to cause severe side effects, can be more effective when appropriately combined with indocyanine green (ICG)-based PDT. Crescenzi et al. conducted a study to examine the effects of combining PDT with low-dose chemotherapy on breast cancer cells. They performed PDT by irradiating human breast adenocarcinoma cells (MCF-7) preloaded with ICG using an IR diode laser source. To find treatment combinations that can reduce the dose of toxic compounds without reducing efficacy, the researchers studied the effects of moderately toxic doses of cisplatin and ICG/PDT, administered individually and in combination, on various metabolic and proliferative parameters of MCF-7 cells. Administered individually, neither treatment was lethal for MCF-7 cells. However, when combined, the reduction in cell viability was much greater than with the treatments administered separately. It was found that ICG/PDT and cisplatin target different phases of the cell cycle (cisplatin is more effective against S-phase cells, while ICG/PDT is more effective against G1-phase cells), and both treatments significantly inhibited DNA synthesis. The study suggested that combining low doses of cytostatic drugs with PDT can be as effective or even more effective than the currently used doses [47].

Zakaria et al. also studied the synergistic cytotoxic effects of low doses of combined chemotherapy and 5-aminolevulinic acid (5-ALA)/PDT on MCF-7 cells compared to high doses of each individual therapy. MCF-7 cells were pre-treated with Doxil^®^ followed by ALA/PDT [48]. Doxil^®^ is a nano-drug formulation of DOX that has been clinically investigated and approved by the FDA. It utilizes the enhanced permeability and retention (EPR) effect to passively target tumors, where DOX is released and made available to tumor cells [49]. Doxil^®^ has been observed to result in higher drug levels in tumor tissue compared to free DOX in multiple cancer models and has shown the ability to reduce cardiotoxicity, a significant side effect of free DOX treatment, in clinical settings [50,51,52]. To evaluate the effect of combined therapy on the viability of MCF-7 cells, 50% of each IC50 of both Doxil^®^ and ALA/PDT were used and compared with the cytotoxicity of each drug individually at their IC50 doses. The results showed a significant reduction in cell viability when both therapies (Doxil^®^ + ALA/PDT) were used at low doses compared to the high doses of each individual therapy. Treatment with combined therapy (Doxil^®^ + ALA/PDT) using half IC50 concentration of each resulted in a significant increase in the total percentage of cell death, reaching 77%. This increase was observed in both apoptotic and necrotic cell populations. In conclusion, the combined therapy using low doses of ALA/PDT and Doxil^®^ exhibited a synergistic cytotoxic effect on MCF-7 cells, which was superior to the treatment with high doses of each individual therapy. This was mainly due to the enhanced intrinsic and extrinsic apoptotic mode of cell death. Therefore, the potential adverse effects of Doxil^®^ or ALA treatment could be minimized while ensuring maximal therapeutic benefit in breast cancer treatment [48].

Aniogo et al. aimed to investigate the effects of DOX and sulfonated zinc phthalocyanine-mediated PDT (ZnPcS-PDT) on MCF-7 cells and determine if combining low doses of the two treatments could serve as a novel approach for treating human breast cancer. The aim was to overcome the dose-limiting effect of DOX. The researchers also investigated the mechanism of cell death resulting from the combined treatment. A study examining the effects of DOX and ZnPcS-PDT on breast cancer cells found that both treatments inhibited the viability of MCF-7 cells in a dose-dependent manner. When the two treatments were combined, a significant reduction in cell viability was observed, with the highest inhibition occurring when 0.5 µM DOX and 1 µM ZnPcS-PDT were used. Moreover, combining DOX and ZnPcS-PDT at lower doses of 0.5 µM DOX and 0.25 µM ZnPcS-PDT and higher resulted in a significant decrease in cell proliferation. To determine cytotoxicity, the amount of lactate dehydrogenase (LDH) released into the culture media was measured after treating MCF-7 cells with DOX and ZnPcS-PDT alone and in combination. The results showed that the highest LDH release occurred with the combination of 0.5 µM DOX and 1 µM ZnPcS-PDT. These findings are consistent with the results of viability and proliferation assays, which indicated that treatment with both DOX and ZnPcS-PDT resulted in a decrease in the viability and biological activity of MCF-7 cells. These findings suggest that combining low doses of DOX with PDT can lead to a significant synergistic effect on MCF-7 cancer cells, resulting in increased apoptosis. Further investigation of this combination therapy in in vivo studies was recommended to determine its potential to reverse multidrug resistance in cancer treatment [53].

Khorsandi et al. investigated the effects of combined treatment with DOX and methylene blue (MB) activated by PDT on triple-negative breast cancer cells. The human breast cancer cell line MDA-MB-231 was exposed to various concentrations of DOX, MB, and the combination therapy of DOX-methylene blue (DOX-MB) under two different conditions: first, DOX followed by MB-PDT, and second, MB-PDT followed by DOX. One of experiments aimed to investigate the effect of DOX on the survival of the breast cancer cell line MDA-MB-231. The study looked at different concentrations of DOX at various incubation times and observed a decrease in cell viability with increasing DOX concentration. Additionally, increasing the treatment time led to an increase in the percentage of cell death. When the cells were treated with MB-PDT and then exposed to DOX, the cell viability decreased to 53% at 0.5 μg/mL and 38% at 1 μg/mL of DOX. In addition, the colony-forming ability of the MDA-MB-231 cells was decreased in the presence of the MB-PDT-then-DOX treatment compared to the DOX alone or control groups [54]. Aniogo et al. conducted a study on the effects of DOX and ZnPc-PDT and found that their combined treatment had a synergistic effect on inhibiting the growth of MCF-7 cells [53]. Similarly, Khorsandi’s study showed that using MB-PDT in combination with DOX, even at low concentrations, was more effective at inducing cancer cell death compared to DOX alone. This study suggested that the combination therapy of MB-PDT–DOX has potential as a strategy for treating triple-negative breast cancer cells [54].

The study by Ahn et al. aimed to evaluate the potential effects of cisplatin in combination with PDT for breast cancer treatment using a breast-tumor-bearing mouse model. The mice were divided into four groups: control, cisplatin, PDT, and combination (cisplatin + PDT). Cisplatin was injected into the abdominal cavity of each mouse 1 h before Photolon^®^ injection, which was then administered 2 h before PDT. The both PDT and combination groups showed a decrease in tumor volume on day 7, indicating that the combination therapy maximized the effectiveness of PDT. The study also measured the levels of lipid peroxidation products in the tumor tissue, and the PDT group showed a 70% increase in thiobarbituric acid reactive substance (TBARS) compared to the control group. The combination group had even higher levels of TBARS (up to 47% more than the PDT group), indicating that the combination therapy increased the oxidative damage of cancer cells. In summary, a combination of low-dose cisplatin and PDT using Photolon^®^ in a breast cancer mouse model led to increased cancer cell necrosis, oxidative damage, and inflammation within tumor tissues. Based on these results, it was proposed that combining cisplatin treatment with PDT may offer additional therapeutic benefits for treating cancerous lesions [55].

Anand et al. studied the use of capecitabine (CPBN) as a non-toxic neoadjuvant for PDT in the treatment of breast cancer, suggesting that it represents a novel approach with significant potential for translation into the clinic. CPBN is a non-toxic precursor of 5-FU, which is selectively converted into the active end product, 5-FU, primarily in cancer cells [56]. To develop a combination approach with CPBN and PDT, the study utilized a mouse breast cancer model, which is a triple-negative breast cancer equivalent to human stage IV, and aimed to better understand the CPBN-PDT approach for possible translation to the clinic for treating localized breast cancer [57,58]. A newer mechanism of PDT has been developed to reduce toxicity and improve tumor selectivity. This mechanism uses a prodrug, ALA, instead of pre-formed photosensitizer [59,60]. ALA is given systemically, orally, or topically and is taken up and enzymatically converted into protoporphyrin IX (PpIX) within mitochondria. PpIX is then activated by strong visible light, generating reactive oxygen species that kill cancer cells [61,62,63]. In this study, female nude mice were injected with luciferase-transgenic murine 4T1 cells in the breast fat pads. The mice were then treated with CPBN followed by ALA administration and PDT. Tumor growth and regression were monitored using bioluminescence imaging from different treatment groups (vehicle, CPBN, vehicle + PDT, and CPBN + PDT). It was demonstrated that pretreatment with CPBN prior to ALA-mediated PDT results in selective enhancement of PpIX levels within 4T1 tumors and improves the outcome of PDT by enhancing tumor cell death. Additionally, histological and immunohistochemical analyses revealed that the CPBN-PDT combination enhances the differentiation state of 4T1 carcinoma cells within tumors. Furthermore, in vivo bioluminescent monitoring showed that the combination approach was more effective than ALA-PDT alone in retarding the growth of the primary tumor and reducing metastatic spread. To summarize, the combination treatment of CPBN and ALA-mediated PDT has shown promising results for the treatment of breast cancer and may have potential for treating other types of cancer as well [56].

### 3.2. PDT with Surgery in Breast Cancer

Breast cancer is typically treated with either breast-conserving surgery (BCS) or mastectomy. BCS combined with post-operative radiotherapy has become the preferred treatment for early-stage breast cancer due to equivalent survival rates to mastectomy and improved QOL. However, complete cancer removal with adequate margins while preserving the natural shape of the breast can be challenging. Tumor location and relative size of the breast can limit tissue removal, and if both goals cannot be achieved, mastectomy may be suggested. Neoadjuvant treatments such as chemotherapy or hormone therapy can downsize the tumor preoperatively, but not all tumors respond to these treatments. The limitations of classical BCS or mastectomy techniques have driven the development of new techniques such as PDT in breast surgery over the past decade [64,65].

Lee et al. conducted a study to compare the therapeutic efficacy of two less invasive treatments, laser ablation and PDT, for early breast cancer. The authors induced breast tumors in 12 mice models and classified them into four groups: a control group (without any treatment, group I), a group treated with laser ablation only (group II), a group treated with PDT only (group III), and a group treated with a combination of laser ablation followed by PDT (group IV). Laser ablation was conducted on the xenografted mice under in vivo conditions, and a biocompatible nanocomplex formulation of methylene blue (nanoMB) solution was injected into the peritumoral site one day later, followed by irradiation for PDT. Group IV showed the most effective result after 15 days and statistical significance compared to groups I and II. Although there was no significant difference between groups III and IV, the mean tumor volume of group IV showed the greatest decreasing pattern. A single procedure with laser ablation did not obtain a negative margin even if cancer necrosis was induced in the center of the tumor. Similarly, breast tumors treated with PDT using nanoMB did not increase in size during the treatment period, but the decrease in tumor volume was negligible. To overcome these disadvantages, the combined method of both procedures showed a therapeutic effect on breast cancer in a mouse model by directly affecting the center of the cancer through laser ablation and managing the tumor margin by PDT [66].

The group led by Banerjee conducted the first clinical study of PDT for the treatment of primary breast cancer. Banerjee et al. supervised a phase I/IIa study to evaluate the efficacy of PDT in the treatment of primary breast cancer. The study enrolled 12 female patients with a new diagnosis of invasive ductal breast cancer and axillary nodal involvement, who were scheduled to undergo mastectomy as their first treatment. The median follow-up was 50 months. During this time, there was only one case of distal metastasis (to the liver) after 3 years, and all patients were alive at the most recent follow-up. In contrast, the control cohort of 14 eligible patients who declined the study had three cases of distant metastases and one case of local recurrence. The study demonstrated that PDT under image guidance is a promising, safe, and minimally invasive treatment for primary breast cancer, which may have a potential role in the management of early breast cancer [67].

### 3.3. PDT with Radiotherapy in Breast Cancer

Radiotherapy is a cancer treatment that utilizes high-energy ionizing rays, either externally or internally, to eliminate cancer cells. Although effective, it can cause short-term side effects like breast swelling and heaviness, sunburn-like skin changes in the treated area, and fatigue. Combining synergistic treatments can offer several advantages, such as enhancing treatment efficacy, reducing the required dosage, minimizing toxicity, and decreasing the likelihood of drug resistance [7].

Several reports indicate that certain photosensitizers can act as radiosensitizers. In the case of breast cancer, numerous in vitro studies have demonstrated a synergistic effect between PDT and ionizing radiation in killing cancer cells. The combined use of indocyanine green, mitoxantrone, Radachlorin^®^, and gallium phthalocyanine chloride at non-toxic doses with light has been highly effective, resulting in almost complete elimination of cancer cell survival. These findings suggest that combining photosensitizer-mediated PDT with ionizing radiation may be more effective than either approach alone, potentially enhancing the therapeutic effect while reducing the radiation dose and associated side effects.

A study was undertaken by Montazerabadi et al. to evaluate the effects of ICG as a sensitizer in both PDT and radiation therapy on the MCF-7 cell line. In this study, the safety of ICG treatment on MCF-7 was evaluated using the MTT assay with different concentrations of ICG [68]. The results indicated that ICG did not exhibit any cytotoxic activity on MCF-7 cells at concentrations up to 100 μM [69]. These findings were consistent with previous studies, such as the one conducted by Mamoon et al., which reported a theoretical calculated half-maximum inhibitory concentration (IC50) of 949.4 μM for ICG [70]. Therefore, ICG is considered a highly safe and biocompatible molecule, making it a suitable photosensitizer for PDT applications, especially for deeper tumors with near-infrared lasers. There was no significant reduction in cell viability at low doses of X-ray radiation, but the maximum reduction was observed at 8 Gy. Interestingly, combining ICG-mediated PDT with X-ray radiation at non-toxic doses showed a potent synergistic effect. The results indicated that a combined treatment with a low radiation dose could enhance the phototoxicity of ICG-mediated PDT on MCF-7 while reducing the side effects of high radiation and light doses. In conclusion, a combination of low doses of ICG-PDT and X-ray radiation had a potent synergistic effect on MCF-7. This combination therapy has the potential advantage of causing less damage to normal cells compared to individual treatment modalities. For future clinical applications, the light dose and ICG dose can be adjusted based on the size and depth of the tumor as well as the patient’s sex, weight, and age [71]. These findings provide a good starting point for the combined treatment with radiotherapy and ICG-PDT applications for breast cancer treatment in clinics. This knowledge is clinically relevant, as patients undergoing PDT treatments are likely to receive ionizing radiation concurrently [68].

The same research team obtained comparable results when using mitoxantrone (MX) as a sensitizer in combination with radiation therapy and PDT on MCF-7 cells. The study found that MX did not induce complete cell death in MCF-7 cells even at higher doses. The researchers performed radiation therapy with doses of 2, 4, 6, and 8 Gy to evaluate the cytotoxic effect of X-ray radiation and the radiosensitizing effect of MX on the cells. The maximum reduction in viability was observed in cells that only received radiation at 8 Gy. However, when X-ray ionizing radiation was combined with MX-mediated PDT, even better results were observed. Notably, cells that received a combination treatment of 2 Gy radiation and MX had a lower percent viability compared to cells that received 8 Gy of radiation without MX. Combining nontoxic doses of the therapies resulted in a potent synergistic effect that decreased the viability of MCF-7 cells. This study found that a combination of low doses of MX (1 µM), light (10 J/cm^2^), and X-ray radiation (4 Gy) had a potent cytotoxic effect on MCF-7 cancer cells, which may be less damaging to normal cells than individual treatment modalities. These findings provide a promising starting point for the clinical application of combined treatment with radiotherapy and MX-PDT in the treatment of breast cancer [72].

Consistent with previous studies, Ghoodarzi et al. reported similar results in 2015. Ghoodarzi et al. investigated the effectiveness of Radachlorin^®^ as a sensitizer in both PDT and radiation therapy on MCF-7. The cells were incubated with Radachlorin^®^ and then treated, in separate groups, with red visible light at two energy densities (6 and 12 J/cm^2^) and 2-Gy X-ray ionizing radiation. The findings revealed that Radachlorin^®^ alone did not have a significant cytotoxic effect. However, when combined with light, Radachlorin^®^ showed a strong cytotoxic effect on MCF-7 cells. Although Radachlorin^®^ could not act as a radiosensitizer, the integration of Radachlorin^®^ with radiotherapy and PDT resulted in a significant reduction in cell viability compared to the control group. The combination therapy of Radachlorin^®^-PDT and X-ray irradiation decreased the viability of MCF-7 cells compared to the group without X-ray irradiation. Combination therapy involving radiation and PDT may be a promising approach for breast cancer treatment that could reduce adverse effects without compromising treatment efficacy [73].

In agreement with previous studies, Mayahi et al. showed consistent results. They investigated the impact of gallium phthalocyanine chloride (GaPcCl) as a radiosensitizer and photosensitizer on MCF-7 cells. The cells were treated with various concentrations of GaPcCl and then exposed to different light doses as well as 2 Gy of X-ray ionizing radiation, either alone or in combination [74]. GaPcCl belongs to the metallophthalocyanine family and has been previously used as an anti-tumor agent by intercalating into DNA [75]. The researchers also assessed the effectiveness of combining GaPcCl-mediated PDT with radiotherapy. Notably, this was the first study to investigate the dual-sensitizing effects of GaPcCl on cancer cells. The cytotoxic effect of GaPcCl without laser activation was also examined, and it was found that DMSO, the solvent for GaPcCl, did not have a significant effect on MCF-7 cell toxicity compared to the control group. When GaPcCl was exposed to a light dose of 2.8 J/cm^2^, cell viability significantly decreased compared to GaPcCl alone. In combination with X-ray irradiation, the combination therapy of GaPcCl-PDT and 2 Gy X-ray irradiation significantly reduced MCF-7 cell survival. According to the MTT assay results, the combination of GaPcCl and X-ray irradiation did not result in significant cell death. However, flow cytometry analysis revealed that the combination induced a substantial increase in cell apoptosis, indicating that GaPcCl could be used as a radiosensitizer. These findings highlight the discrepancy between the two assays, suggesting that further experiments are needed to confirm the radiosensitizer potential of GaPcCl. In conclusion, the combinational effect of PDT and radiotherapy was found to be significantly more effective in killing MCF-7 cells compared to PDT alone. Interestingly, the flow cytometry results showed a significant increase in the percentage of late apoptotic cells when the GaPcCl-PDT group was exposed to 2 Gy X-rays, indicating the potential of GaPcCl as a photosensitizer in MCF-7 cells. Thus, combining GaPcCl-mediated PDT with radiation therapy may be a promising strategy for the treatment of breast cancer [74].

Shrestha et al. conducted a study on the use of copper-cysteamine (Cu-Cy) nanoparticles, which are designed to be targeted to tumors, for X-ray-induced PDT. Their findings confirmed the effectiveness of Cu-Cy nanoparticles as a photosensitizer when activated by radiation and suggested that these nanoparticles could be good candidates for PDT in deep-seated tumors when combined with X-rays and conjugated to a tumor-targeting molecule [76]. The study also explored the use of a pH-low insertion peptide (pHLIP) to actively target the nanoparticles to low pH tumors in the future [77,78,79,80]. In this study, mice were divided into six treatment groups to investigate the effects of pHLIP-Cu-Cy nanoparticles and radiation on tumor size reduction: (i) pHLIP-Cu-Cy nanoparticles + radiation, (ii) Cu-Cy nanoparticles + radiation, (iii) PBS + radiation, (iv) pHLIP-Cu-Cy nanoparticles, (v) Cu-Cy nanoparticles, and (vi) PBS (control). The results showed a statistically significant reduction in tumor size under X-ray activation of pHLIP-conjugated Cu-Cy nanoparticles compared with X-ray alone in mouse tumors. In summary, the study found that the use of pHLIP-conjugated Cu-Cy nanoparticles resulted in a statistically significant reduction in tumor size when compared to plain Cu-Cy nanoparticles or radiation therapy alone. The difference between radiation therapy and pHLIP-conjugated Cu-Cy nanoparticles versus radiation therapy and plain Cu-Cy nanoparticles was also statistically significant, indicating that pHLIP conjugation improved the effectiveness of the treatment. Additionally, Cu-Cy nanoparticles can be activated by both X-rays and light, making them useful in treating both shallow and deep tumors [81,82]. Overall, the study suggested that pHLIP-conjugated Cu-Cy nanoparticles can enhance radiation therapy and improve tumor size reduction in male and female mice [76].

**Table 2:** Combined therapies with PDT in breast cancer.

Combined Therapy		Photosensitizer	Dose	Light Source	Study Type	Ref.
Chemotherapy	cisplatin (8 μM)	ICG	20 μM	805 nm (25 J/cm^2^)	in vitro	[47]
	doxorubicin (9.925 μM)	5-ALA	0.55 mM	633 nm (0.25 W)	in vitro	[48]
	doxorubicin (0.5 µM)	sulfonated zinc phthalocyanine (ZnPcS)	0.25 µM	681.5 nm (5 J/cm^2^)	in vitro	[53]
	doxorubicin (0.5 μg/mL)	methylene blue	15 μg/mL	660 nm (3 J/cm^2^)	in vitro	[54]
	cisplatin (3 mg/kg)	Photolon^®^	2.5 mg/kg	660 nm (80 J/cm^2^)	in vivo	[55]
	capecitabine (600 mg/kg/day)	5-ALA	200 mg/kg	633 nm (100 J/cm^2^)	in vivo	[56]
Surgery	laser ablation followed by PDT	methylene blue nanocomplex	10.05 mg/mL	655 nm (0.2 J/cm^2^)	in vivo	[66]
	mastectomy after PDT	verteporfin	0.4 mg/kg	690 nm (20, 30, 40, 50 J/cm^2^)	clinical	[67]
Radiotherapy	X-ray (4 Gy)	ICG	50 µM	730 nm (60 J/cm^2^)	in vitro	[68]
	X-ray (4 Gy)	mitoxantrone	1 µM	660 ±10 nm (10 J/cm^2^)	in vitro	[72]
	X-ray (2 Gy)	Radachlorin^®^	2.5 μg/mL	660 nm (12 J/cm^2^)	in vitro	[73]
	X-ray (2 Gy)	gallium phthalocyanine chloride (GaPcCl)	100 μg/mL	660 nm (2.8 J/cm^2^)	in vitro	[74]
	X-ray (5 Gy)	pH-low insertion peptide (pHLIP) conjugated copper-cysteamine (Cu-Cy) nanoparticles	16 μg	-	in vivo	[76]

## 4. PDT in Cholangiocarcinoma and Combination Therapies

Cholangiocarcinoma is a rare and aggressive cancer that arises from the bile ducts, which are the tubes that carry bile from the liver to the intestine. Cholangiocarcinoma can develop at any location along the biliary tree. Intrahepatic cholangiocarcinoma, perihilar cholangiocarcinoma, and distal cholangiocarcinoma differ in their tumor characteristics, and each type has its own staging system established by guidelines. Perihilar cholangiocarcinoma accounts for 60% to 70% of all cases of cholangiocarcinoma, intrahepatic cholangiocarcinoma accounts for 5% to 10%, and distal cholangiocarcinoma accounts for 20% to 30% [83]. The Bismuth classification system categorizes hilar cholangiocarcinoma into four different types based on their location in the main branches: type I tumors are limited to the common bile duct with a distance of more than 2 cm from the confluence, type II tumors involve the confluence, type III tumors involve either the right (IIIa) or left (IIIb) hepatic duct, and type IV tumors extend to both ducts or are multifocal in location [84]. Unfortunately, the disease is associated with significant morbidity and mortality, with an average 5-year survival rate of 5% to 10% [85]. Surgical resection is the only curative option, but more than 80% of patients present with an advanced and unresectable stage, leading to a median survival of 3–6 months from the time of diagnosis. Palliative treatments such as chemotherapy, radiation therapy, and biliary drainage (endoscopic or percutaneous) have been attempted, but no definitive survival benefit has been established. The prognosis and treatment options for this malignancy depend on the stage of the disease and the location of the tumor. In the early stages of cholangiocarcinoma, surgical resection is the preferred treatment, as it offers the best chance of a cure. However, many patients are diagnosed at a late stage when the cancer has already spread beyond the bile ducts. In these cases, endoscopic biliary drainage, chemotherapy, and radiation therapy are commonly used to manage the disease. Liver transplantation can also be a curative option for patients with perihilar or distal cholangiocarcinoma who meet specific criteria for transplantation. However, the scarcity of donor organs and the aggressive nature of the disease make it a challenging option. For patients who are not candidates for surgery or transplantation, local treatments such as hepatic artery-based therapies, brachytherapy, and PDT may be used to control the disease and improve symptoms. These treatments target the tumor directly and can be effective in prolonging survival and improving QOL. PDT is an especially promising new treatment option for nonresectable cholangiocarcinoma. PDT offers symptomatic relief and prolonged survival with relatively few complications. Overall, the management of cholangiocarcinoma requires a multidisciplinary approach and individualized treatment plans based on the patient’s stage of disease, location of the tumor, and overall health status [86,87]. In Table 3, the combined therapies involving PDT in cholangiocarcinoma are summarized.

### 4.1. PDT with Chemotherapy in Cholangiocarcinoma

The combination of intravenous systemic chemotherapy with gemcitabine and cisplatin is considered the standard of care for palliative treatment of advanced biliary tract cancer [88]. PDT has been shown to provide benefits in treating the targeted area in patients with bile duct carcinoma (BDC) who are receiving chemotherapy or adjuvant chemotherapy after surgery [89]. Therefore, PDT is a promising treatment option to enhance the effectiveness of conventional anticancer chemotherapy and brachytherapy in the management of BDC.

Nonaka et al. conducted an experiment to examine the treatment effects of PDT using a new photosensitizer called talaporfin sodium (Laserphyrin^®^) in combination with conventional anticancer drug treatments in a BDC cell line (NOZ) both in vitro and in vivo. The study aimed to assess the synergistic effect of the combined treatment. The photosensitizer properties of talaporfin sodium (NPe6, Laserphyrin^®^; TPS) were studied in vitro. PDT was performed for 24 h on a mixture of NOZ cells that had been previously treated with an anticancer drug. The NOZ cell mixture was exposed to TPS and then irradiated. The study investigated the effect of combining PDT with various anticancer drugs (cis-diamminedichloroplatinum, oxaliplatin, gemcitabine, and 5-FU) on the viability of NOZ cells. Results showed that PDT alone and PDT combined with anticancer drugs significantly reduced cell viability compared to the control group. Among the combinations tested, co-administering PDT with gemcitabine and oxaliplatin showed the lowest NOZ cell viability in vitro. In animal experiments, Laserphyrin^®^ was injected intraperitoneally into mice. The interval between the Laserphyrin^®^ injection and light exposure was 2 h [90]. The histological analysis showed the effects of co-administering PDT with oxaliplatin and gemcitabine on tumor necrosis, cell apoptosis, proliferating cell nuclear antigen labeling index (PCNA LI), and percentage of the VEGF-immunopositive area (PVIA) (%). The control group (no treatment) did not show any tumor necrosis, while the PDT alone and PDT with oxaliplatin and gemcitabine groups had a significantly higher tumor necrotic area than the oxaliplatin and gemcitabine treatment group. However, there was no significant difference in the necrotic areas between the PDT alone and PDT with anticancer drugs group. Compared to the control group, the combination of oxaliplatin and gemcitabine, PDT alone, or PDT with these anticancer drugs had a significantly higher percentage of cell apoptosis. There was no significant difference in the apoptosis-positive percentage between the PDT alone and anticancer drug alone groups. PCNA LI was significantly lower in all other groups besides the control group. PDT alone or PDT with oxaliplatin and gemcitabine showed a significantly lower PCNA LI than the oxaliplatin and gemcitabine alone. Furthermore, PDT combined with oxaliplatin and gemcitabine showed a significantly lower PCNA LI than PDT alone. In comparison to the control group, PVIA was significantly lower in all other groups. PVIA of the PDT combined with oxaliplatin and gemcitabine was significantly higher than the coadministration of oxaliplatin and gemcitabine alone or PDT alone. The results showed that the combined treatment of PDT and a single dose of an anticancer drug led to lower tumor viability compared to PDT treatment alone. Moreover, the combination of two drugs had an increased synergistic cytocidal effect. The in vivo study demonstrated a more potent apoptotic induction, lower proliferative activity, and higher VEGF expression when administering PDT combined with anticancer drugs compared to chemotherapy alone or PDT alone treatments. PDT has a direct cytotoxic effect on tumors and indirectly affects the tumor microenvironment, inducing apoptosis, an inflammatory reaction, tumor-specific and/or nonspecific immune reactions, and microvasculature damage [91,92,93]. Although the tumor necrotic area in the PDT alone and the PDT with anticancer drugs groups was similar, the apoptosis-positive area was higher in the group receiving PDT with chemotherapy treatment than in PDT alone. PDT using Laserphyrin^®^ appears to have potential against BDC cells in preclinical models and represents an important area for future clinical trials [94].

In a study by Hong et al., the long-term outcomes of PDT combined with systemic chemotherapy were compared to those of PDT alone in patients with advanced hilar cholangiocarcinoma. Patients were divided into two groups, A and B, according to their treatment modality. Group A consisted of 16 patients who received PDT combined with gemcitabine, with or without cisplatin, while 58 patients in group B received PDT alone. All patients included in the study were considered unresectable according to accepted criteria [95,96]. Two groups of patients with advanced hilar cholangiocarcinoma were compared, with the only significant differences being in lymph node metastasis and frequency of ERCP in the PDT group. Lymph node metastasis was more common in group A than in group B. Additionally, the frequency of ERCP was higher in group A than group B. However, there were no differences in age, sex, TNM stage, bismuth type, CA 19–9 level, or pre-PDT albumin level between the two groups. In group A, nine patients were treated with gemcitabine alone, and six received gemcitabine with cisplatin. Among patients who received PDT, 70.3% received only one dose of PDT and the rest received more than two. The study found that the survival of the PDT with chemotherapy group tended to be greater than the PDT alone group. The median survival was 17.9 months for PDT with chemotherapy and 11.1 months for PDT alone. Overall, the addition of PDT to systemic chemotherapy appears to be a promising new approach to treating advanced bile duct cancer [97].

Park et al. conducted a prospective, randomized phase II trial to compare the effectiveness of PDT plus S-1 and PDT alone for the treatment of locally advanced, unresectable hilar cholangiocarcinoma (UHC). S-1 is an oral prodrug containing tegafur, 5-chloro-2,4-dihydroxypyridine, and potassium oxonate. Patients with UHC were randomly assigned to either PDT plus S-1 or PDT alone in a 1:1 ratio. The primary endpoint was overall survival, and the secondary endpoints were progression-free survival, complications, re-intervention rate, and QOL. This was the first prospective randomized trial to investigate the role of PDT plus systemic chemotherapy in the management of UHC. The primary analysis of this trial was conducted after a median follow-up of 13 months (±7.9 months), and the final analysis was event-driven, performed 12 months after the last patient was enrolled. The results showed a significant increase in overall survival in the PDT plus S-1 group compared to the PDT alone group. The median overall survival in the PDT plus S-1 group was 17 months, compared to 8 months in the PDT alone group. Patients who received PDT plus S-1 were 64% less likely to die at any time than those who received PDT alone. In terms of secondary endpoints, patients in the PDT plus S-1 group had a longer period of progression-free survival compared to those in the PDT alone group (median 10 months versus 2 months). There were no significant differences between the two groups in terms of adverse events and QOL. In conclusion, PDT plus S-1 demonstrated promising efficacy in terms of 1-year survival rate, overall survival, and progression-free survival compared to PDT alone, without increasing the rates of serious adverse events in patients with UHC. The findings suggest that PDT with S-1 may provide clinical benefit for patients with UHC [98].

Wentrup et al. conducted a retrospective analysis of 68 patients with nonresectable hilar cholangiocarcinoma (NCC). The patients were divided into two groups: PDT alone (PDT-M) (*n* = 35) and PDT plus chemotherapy (PDT-C) (*n* = 33). None of the patients had previously received chemotherapy or PDT for cholangiocarcinoma at the start of the study. All NCC patients underwent endoscopic stent placement after PDT, with regular stent changes after 3 months or earlier if there were any signs of cholangitis or stent occlusion. The study found that the mean overall survival in all NCC patients was 483 days. The PDT-C group showed a significantly longer mean survival of 520 days compared to the PDT-M group’s mean survival of 374 days. Furthermore, the 1-year survival rate was significantly higher in the PDT-C group (87.9%) compared to the PDT-M group (51.4%). When the different chemotherapy protocols were analyzed in the PDT-C group, the combination therapy with gemcitabine showed the longest overall survival. Cholangitic complications were observed in 76.5% of patients, and there was no significant difference between the two groups regarding the occurrence of cholangitic complications. In summary, the study suggested that combining repeated PDT with a gemcitabine-based combination chemotherapy could provide a significant survival benefit, which is in agreement with a recent observation from Korea [97]. The study also showed that chemotherapy does not increase the risk of cholangitis. The preferred chemotherapy protocol in combination with PDT could be platinum with gemcitabine [99].

Gonzalez-Carmona et al. conducted a study to assess the efficacy of PDT in combination with systemic chemotherapy for advanced extrahepatic cholangiocarcinoma. Patients were divided into three groups: PDT plus chemotherapy, PDT alone, or chemotherapy alone. The PDT chemotherapy group had a significantly longer median survival of 20 months compared to 15 months in the PDT alone group and 10 months in the chemotherapy alone group. The PDT chemotherapy group also showed higher 1-year (77.7% vs. 34.1%) and 2-year survival (27.5% vs. 17.5%) compared to the chemotherapy alone group. This study supports previous findings by Hong et al. [21] and Wentrup et al. [99] that combining PDT with chemotherapy can prolong median survival in patients with metastatic cholangiocarcinoma by four to seven months. The results suggest that local control of the tumor is at least as important as systemic treatment in managing metastatic disease. However, the combination of PDT with chemotherapy did not result in more adverse events than chemotherapy or PDT alone, and cholangitis recurrence and severity were evenly distributed among the three groups. In summary, this study provided evidence for the efficacy of PDT in controlling advanced cholangiocarcinoma, and the combination with chemotherapy resulted in a significantly longer overall survival compared to chemotherapy alone. The use of PDT was also significantly correlated with longer survival [100].

### 4.2. PDT with Surgery in Cholangiocarcinoma

The only potential cure for cholangiocarcinoma is complete surgical resection. However, the majority of patients are deemed ineligible for surgical resection due to metastatic or locally advanced disease at the time of diagnosis. This circumstance leads to a poor prognosis of approximately 3 months without intervention and 4–6 months with palliative biliary decompression [101]. Even after curative (R0) resection, the 5-year survival rate remains low at only 30–40% [102,103,104]. Therefore, the primary goal of successful palliation of biliary obstruction is reducing morbidity and mortality in these patients [105,106]. For patients diagnosed with UHC, the current standard of care for managing the disease is palliative treatment, which now includes an endoscopic approach. However, it is important to note that the primary goal of biliary stent placement is to delay the occurrence of bile duct obstruction rather than reducing tumor growth. Nevertheless, PDT in conjunction with biliary stents is commonly utilized for palliation of jaundice and to improve overall survival [107,108,109,110,111].

#### 4.2.1. PDT with Resection in Cholangiocarcinoma

Wiedmann et al. conducted a study on seven patients who had advanced proximal bile duct carcinoma. These patients were treated with PDT and underwent surgery after a median period of 6 weeks. All patients underwent liver resections on either the right, left, or both sides. At the time of the study, five patients were still alive without recurrence of hilar cholangiocarcinoma. Two patients developed clinically proven tumor recurrence and died of tumor progression. Late complications included duodenal stenosis, upper digestive tract bleeding, and cholangitis, which were not seen with neoadjuvant PDT treatment. In conclusion, neoadjuvant PDT for hilar cholangiocarcinoma is a low-risk procedure that efficiently destroys the tumor within a superficial 4 mm layer. Post-operative complications are similar to series without neoadjuvant PDT. In the future, neoadjuvant PDT may achieve curative resection of hilar cholangiocarcinoma in a higher percentage of patients and reduce the rate of local disease recurrence after curative resection. Additionally, the development of new photosensitizers for PDT that can penetrate tumoricidal tissue to at least a depth of 8 mm is necessary [112].

Nanashima and colleagues conducted a study to evaluate the effectiveness of adjuvant PDT in controlling remnant tumors in the hepatic duct stump and treating severely obstructed bile ducts in eight patients who underwent surgical resection for extrahepatic or intrahepatic BDC. Of the eight patients, five had extrahepatic BDC, two had intrahepatic cholangiocarcinoma, and one had ampullary carcinoma. One patient died due to unrelated causes, while another patient remained tumor-free for 30 months after PDT before developing pleural and bone metastases. Four patients had no tumor recurrence, and one patient had a local remnant tumor on CT scan but no re-occlusion of the biliary tract for 20 months. One patient experienced biliary re-occlusion eight months after PDT. The study concluded that PDT is a safe and effective option for local tumor control in patients with resectable and non-resectable bile duct carcinomas with remnant tumor cells in the hepatic duct after surgical resection or tumor recurrence following curative surgical resection. Adjuvant PDT therapy could therefore lead to improved survival outcomes for such patients [89].

#### 4.2.2. PDT with Stent in Unresectable Cholangiocarcinoma

There have been multiple studies investigating the use of combination PDT with stent insertion for the treatment of unresectable hilar cholangiocarcinoma.

Kahaleh et al. conducted a study comparing survival in patients with unresectable cholangiocarcinoma who underwent ERCP with PDT and stent placement versus those who underwent ERCP with stent placement alone. Between December 2001 and November 2006, 64 consecutive patients with cholangiocarcinoma were referred to their institution, of which 16 (25%) underwent resection, and the remaining 48 were palliated with endoscopic biliary stents. Of these, 19 received PDT after its availability in December 2004, while the remaining 29 (60%) underwent ERCP with stenting alone. Both groups showed a significant decrease in bilirubin levels after treatment, but there was no significant difference in the degree of decrease between the two groups. Among the 19 patients who received concomitant extracorporeal radiation, 9 were in the PDT group and 10 were in the stent-only group. Kaplan–Meier survival analysis showed a statistically significantly longer survival time in the PDT group (mean of 16.2 ± 2.4 months) compared to the stent-only group (mean of 7.4 ± 1.6 months). In the PDT group, the 3-, 6-, and 12-month mortality rates were 0%, 16%, and 56%, respectively, while the corresponding mortality rates in the stent-only group were 28%, 52%, and 82%, respectively [107]. The difference was statistically significant at 3 and 6 months but not at 12 months. These results were consistent with those of Ortner et al. [108], who also observed a significant increase in median survival after PDT (16.4 months) compared to patients receiving stent placement alone (3.3 months). In conclusion, ERCP with PDT appears to improve survival in unresectable cholangiocarcinoma compared to ERCP with biliary stenting alone [107].

Quyn et al. conducted a prospective clinical cohort study to evaluate the efficacy of radical curative surgery, standard palliative therapy (stent ± chemotherapy), and a novel palliative therapy (stent ± Photofrin^®^-PDT) in 50 consecutive patients with hilar cholangiocarcinoma. Of the 50 patients, 10 were suitable for curative resection (cohort 1), while 40 patients with irresectable disease were stratified into cohort 2 (stent ± chemotherapy (*n* = 17)) and cohort 3 (stent ± PDT (*n* = 23)). The 1-year survival rate was 80% in cohort 1, 12% in cohort 2, and 75% in cohort 3. The mean survival in cohort 1 was 1278 days, and the median survival had not yet been reached. In cohort 2, the mean and median survival were 173 days and 169 days, respectively, similar to that reported by others for Bismuth type III-IV hilar cholangiocarcinoma. In cohort 3, the mean and median survival were 512 and 425 days, respectively, with the median survival being substantially longer than that observed in cohort 2, and this difference in survival was highly significant [113]. Previous studies have shown that surgical biliary bypass or biliary stenting results in a median survival of approximately 4–10 months [103,106,114,115,116,117,118]. In this study, the median survival for patients in cohort 2 who received standard treatment was also 6 months, which is consistent with the findings of previous studies. In conclusion, this prospective clinical cohort study showed that radical surgery and palliative Photofrin^®^-PDT are associated with increased survival in patients with hilar cholangiocarcinoma [113].

Cheon et al. conducted a retrospective analysis to identify patients who were diagnosed with hilar cholangiocarcinoma. Of these, 72 patients (31%) were treated with PDT in addition to biliary stenting without chemoradiation (Group A), and 71 patients (31%) were treated with endoscopic stenting alone (Group B). PDT was offered as a palliative modality to patients with unresectable hilar cholangiocarcinoma who consented to PDT with either PTCS or ERCP. A total of 18 patients in Group A and 15 patients in Group B underwent biliary drainage with plastic stent insertion followed by the placement of a self-expanding metal stent. Subgroup analyses of patients with metal stents showed that stent patency was longer in Group A than in Group B, with a median stent patency of 215 days in Group A and 181 days in Group B. Median survival was 9.8 months in Group A and 7.3 months in Group B, with 1-, 2-, and 3-year survival rates of 39.4%, 13.9%, and 4.1% in Group A and 26.6%, 9.4%, and 0% in Group B, respectively. The study found that survival was significantly longer in patients who received multiple PDT treatments than in those who underwent a single PDT treatment in univariate analysis. Overall, PDT with stenting resulted in longer survival than stenting alone, and survival was improved by providing early PDT after diagnosis and multiple PDT treatments. Furthermore, metal stent patency was longer in patients receiving PDT [119].

Li et al. conducted a retrospective cohort study on 62 patients with unresectable hilar cholangiocarcinoma. The study aimed to investigate the clinical efficacy and safety of ERCP- or PTCS-directed PDT combined with stent placement for this condition, with 30 patients receiving the combined treatment (PDT + stent group), and 32 patients receiving only stent placement (stent-only group). The median survival time was longer in the PDT + stent group than in the stent-only group (14.2 months vs. 9.8 months). The PDT + stent group also demonstrated significantly greater survival rates at 12 months, 18 months, and 24 months compared to the stent-only group, but not at 6 months. When assessing survival in the absence of recurrence, the PDT + stent group still showed longer median survival time than the stent-only group (13.0 months vs. 9.3 months). For analysis of QOL, the overall scores on the FACT-HEP scale did not differ between the PDT + stent and stent-only groups before treatment and 3 months post-treatment, but were significantly higher in the PDT + stent group at 6, 9, and 12 months post-treatment compared to the stent-only group. There were no significant differences in the incidences of specific postoperative adverse events between the PDT + stent and stent-only groups. The goals of cancer treatment include not only prolonged survival and improved survival rate but restoration of QOL through physical, psychological, and social recovery. In conclusion, PDT can improve the survival time and long-term QOL [120].

**Table 3:** Combined therapies with PDT in cholangiocarcinoma.

Combined Therapy		Photosensitizer	Dose	Light Source	Study Type	Ref.
Chemotherapy	oxaliplatin (10 mg/kg), gemcitabine (50 mg/kg)	Laserphyrin^®^	5 mg/kg	664 ± 2 nm (60 J/cm^2^)	in vitro in vivo	[94]
	gemcitabine, ± cisplatin	porfimer sodium	2 mg/kg	-	clinical	[97]
	S-1	-	-	-	clinical	[98]
	gemcitabine, platinum	Photofrin II^®^	2 mg/kg	630 nm (180 J/cm^2^)	clinical	[99]
	-	Photosan^®^ Photofrin^®^ Foscan^®^	1.5–2.5 mg/kg, 2 mg/kg, 0.04 mg/kg		clinical	[100]
Surgery	resection	Photofrin^®^	2 mg/kg	630 nm (242 ± 20 J/cm^2^)	clinical	[112]
	resection	Photofrin^®^	2 mg/kg	630 nm (50–100 J/cm^2^)	clinical	[89]
	stent	Photofrin^®^	2 mg/kg	633 ± 3 nm (180 J/cm^2^)	clinical	[107]
	stent	Photofrin^®^	2 mg/kg	630 nm laser (180 J/cm^2^)	clinical	[113]
	stent	Photofrin II^®^	2 mg/kg	633 ± 3 nm (180–200 J/cm^2^)	clinical	[119]
	stent	hematoporphyrin	2 mg/kg	630 nm (250 J/cm^2^)	clinical	[120]

## 5. PDT in Cervical Cancer and Combination Therapies

Cervical cancer, derived from the cervix, is the third- or fourth-most common cancer in women worldwide and the most prevalent in less developed countries [121,122]. In over 90% of cases, it is caused by human papillomavirus infections [122,123]. Treatment options for cervical cancer include surgery, radiotherapy, and chemotherapy based on the stage of the disease [124,125]. While radical hysterectomy is considered one curative treatment, it has a recurrence rate of over 10% and a less than 5% 5-year survival rate. Adjuvant therapy, either before or after surgery, has been attempted for primary or recurrent cervical cancer [126,127,128,129,130,131,132,133]. In FIGO stage IA1 cervical cancer, the risk of lymph node metastasis is less than 1% [134]. Treatment options for women wishing to preserve fertility can include conization. For those who do not desire fertility preservation, a simple extrafascial hysterectomy can be performed. In stage IA2, the risk of lymph node involvement increases to up to 8% [135]. The standard treatment for stage IA2 has traditionally been a radical hysterectomy and bilateral pelvic lymphadenectomy [136,137]. The preferred treatment modality for stage IB1 cervical tumors is radical hysterectomy with pelvic and para-aortic lymphadenectomy. These tumors are usually visible to the naked eye and are smaller than 4 cm in diameter. Alternatively, primary radiotherapy can be considered as a treatment option. Surgery offers several advantages over radiotherapy, including the preservation of vaginal function, shorter treatment duration, and the avoidance of radiation-induced menopause in younger patients, which allows for more options for fertility treatments [138,139]. The risk of ovarian metastases is low, and it is standard practice to conserve normal-appearing ovaries in women under 45 years of age [140,141,142]. For stage IB2 cervical cancers, treatment options include radical hysterectomy with pelvic and para-aortic lymphadenectomy followed by adjuvant radiotherapy or chemo-radiotherapy (usually cisplatin-based). Another option for stage IB2 and other stages is external beam pelvic radiotherapy and vaginal brachytherapy followed by a simple hysterectomy. Definitive concurrent chemo-radiotherapy is also a treatment option for certain cases [143]. However, for stages IIB to IVA, non-surgical treatment is preferred, and the specific approach depends on the availability of radiotherapy facilities in the local area. Definitive chemoradiotherapy is considered the standard of care for stages IIB to IVA cervical cancer. This approach is favored because surgery alone is unlikely to provide a curative outcome, and combining radical surgery with chemoradiotherapy increases the risk of adverse events and long-term complications [144]. In Table 4, the combined therapies with PDT in cervical cancer are summarized.

### 5.1. PDT with Chemotherapy in Cervical Cancer

Platinum-based adjuvant chemotherapy, such as cisplatin, is commonly used as a primary treatment for several types of solid tumors, including testicular, ovarian, cervical, bladder, head and neck, colorectal, and small cell lung cancers [145,146,147]. In the case of early-stage cervical cancer, cisplatin has been proven effective in controlling or delaying tumor growth [148]. For advanced or recurrent cervical cancer, the current standard of care involves combination cisplatin-based chemotherapy. In many cases of advanced cervical cancer, a combination of cisplatin and docetaxel is used in concurrent chemoradiotherapy, which has shown a more pronounced sensitizing effect. Carboplatin, although less extensively studied and understood than cisplatin in cervical carcinoma, is often used due to its convenience as an outpatient regimen. When combined with paclitaxel, carboplatin allows for a shorter 3-h infusion, facilitating outpatient treatment. Traditionally, paclitaxel has been administered as a 24-h infusion when combined with cisplatin to reduce neurologic toxicity, necessitating the use of an outpatient infusion pump or hospitalization for each cycle. The Gynecologic Oncology Group 158 trial compared cisplatin and paclitaxel to carboplatin and paclitaxel in ovarian cancer. The combination of carboplatin and paclitaxel was shown to be equally effective as cisplatin and paclitaxel and, due to its outpatient administration, has become the accepted standard of care [149]. Moore’s study also suggested that using carboplatin and paclitaxel instead of cisplatin and paclitaxel may be a therapeutically equivalent option in cervical cancer [150]. However, it is important to note that the short lifespan of platinum-based chemotherapy and the development of tumor resistance are significant drawbacks [151,152]. Consequently, researchers are exploring new strategies that combine platinum-based adjuvant chemotherapy with other therapies to mitigate adverse effects and achieve more favorable clinical outcomes [153].

Wei et al. conducted a study on the use of Hela cells as an in vitro model for human cervical cancer. They first evaluated the effects of single treatments with 5-ALA/PDT, cisplatin, and a combination treatment of 5-ALA/PDT and cisplatin on cell viability. Subsequently, they investigated the mechanism underlying the synergistic anticancer activity of the combined therapies by observing changes in cell apoptosis and the expression of the p53 signaling pathway. The results demonstrated that the combination treatment of 5-ALA/PDT and low-dose cisplatin effectively inhibited cell proliferation in Hela cells. Additionally, 5-ALA/PDT combined with relatively low-dose cisplatin had a greater potential to induce apoptosis in Hela cells, and increasing the concentration of cisplatin in the combination treatment group did not improve the apoptotic rate. The expression of p53, p21, Bcl-2, and Bax in Hela cells after 5-ALA/PDT, cisplatin, and combination treatment was also assessed. The combination treatment resulted in a significant increase in p53 expression compared to single treatments, and p21 expression was significantly higher compared to cisplatin treatment alone. This indicated that PDT in combination with low-dose cisplatin can induce cell apoptosis to a greater extent through the activation of the apoptosis-related protein p53 compared to each single treatment. The expression pattern of Bax and Bcl-2 following the combination treatment showed a similar tendency but with a stronger effect compared to 5-ALA/PDT alone. This suggests that the combination treatment significantly enhances the molecular functional changes in the p53 signaling pathway induced by cisplatin treatment in Hela cells. These findings have the potential to inform new treatment strategies and contribute to reducing the required dosage of anticancer agents. By reducing the amount of cisplatin and photosensitizer needed for therapy, it may be possible to minimize the toxicity and side effects experienced by patients. Thus, their findings suggested that the administration of 5-ALA/PDT in combination with low-dose cisplatin could be an effective and feasible therapy for cervical cancer [154].

Ahn et al. conducted a study on concurrent low-dose carboplatin/Photofrin^®^-PDT (ccPDT) and its efficacy in promoting relapse-free complete tumor regression as a fertility-preservation therapy in cervical or endometrial cancer patients. The study demonstrated that ccPDT led to relapse-free periods of more than 3 years in treated patients while also preserving fertility and enabling successful pregnancies and deliveries, as previously reported in their clinical efficacy study [155]. The cytotoxicity of carboplatin and Photofrin^®^-mediated PDT (PF-PDT) was investigated, and the results revealed that up to 100 µM carboplatin did not induce significant cytotoxicity in HeLa cells. However, when treated with 100 µM carboplatin in combination with varying light doses and 20 µM Photofrin^®^ (ccPDT), there was a significant decrease in cell viability compared to each individual treatment alone. The relative cytotoxicity ratio of the combined treatment to PF-PDT alone gradually with increasing light doses. Furthermore, the combined treatment of ccPDT resulted in an increased apoptotic effect compared to individual treatments. PF-PDT alone mainly induced necrotic death, while ccPDT showed enhanced apoptosis. In terms of reactive oxygen species (ROS) production, ccPDT demonstrated enhanced ROS production compared to the untreated or PF-PDT alone groups. The generation of hydroxyl radicals (OH) was highly enhanced in the combined treatment group compared to PDT alone, with a further increase observed with higher light doses. Additionally, the combined treatment group showed a gradual increase in the production of H_2_O_2_. Choi’s group aimed to demonstrate the enhanced production of hydroxyl radicals and the related apoptotic effects in the ccPDT regimen. Apoptosis was not significantly induced by CBP alone, but it was greatly enhanced by the accumulated OH [156,157], indicating a synergistic effect of ccPDT. Therefore, the therapeutic enhancement observed in low-dose CBP-based ccPDT may be attributed to the synergistic enhancement of OH, leading to apoptosis-based cellular death while minimizing side effects by reducing the effective dosage of carboplatin [158].

De Freitas et al. conducted a study to assess the impact of PDT and cisplatin, both individually and in combination, on cervical carcinoma cells infected and not infected with HPV16 in vitro. They utilized three cell lines: SiHa (cervical carcinoma infected with HPV16), C-33 A (cervical carcinoma not infected with HPV), and HaCaT (spontaneously immortalized human keratinocytes). The researchers employed two different photosensitizers, photogem (PG) and methylene blue (MB), along with cisplatin [159]. Increasing the light dose during PDT led to an augmented phototoxic effect, with SiHa cells exhibiting greater sensitivity compared to the other cell lines. The IC50 for MB-PDT could not be determined for C-33 A cells, indicating potential resistance to MB-PDT compared to SiHa and HaCaT cells. Comparing MB-PDT with PG-PDT, a light dose dependency on the phototoxic effect was observed for PG-PDT, which exhibited higher cytotoxicity than MB-PDT. Among the cell lines, C-33 A was the most sensitive at higher PG concentrations, while SiHa and HaCaT showed similar results. The combination of PDT with cisplatin was highly effective in reducing the IC50 of cisplatin in all cell lines, regardless of the incubation protocol. PDT was tested in two different conditions. In the first condition, either MB or PG was applied before incubation with cisplatin. In the second condition, the PDT protocol was applied after incubation with cisplatin. For MB, performing PDT before cisplatin treatment for 12 or 24 h produced better results compared to irradiating the cells after incubation with cisplatin. However, when cells were incubated with cisplatin for 6 h, the treatment order did not result in any differences. When the photosensitizer was changed to photogem, the incubation time affected the results. PG-PDT resulted in significant cell death when cells were treated with cisplatin for 6 or 12 h before PDT. However, for a 24-h incubation time, the best reduction in cellular viability was observed when PDT was applied before cisplatin. Overall, both photosensitizers were effective in reducing cancer cell viability under the tested conditions. PG-PDT showed higher selectivity for cancer cells at low photosensitizer and light concentrations, while MB-PDT demonstrated a slight advantage by inducing greater phototoxicity in SiHa cells and lesser phototoxicity in HaCaT cells, even with the highest light dose. The presence of HPV16 in SiHa cells may contribute to their increased sensitivity to MB-PDT, although the exact mechanisms are not yet elucidated. Considering that most cervical cancer cases are caused by HPV infection, these findings were particularly relevant. The study indicated that concentrations above 166 μM of cisplatin, which is 12.5 times higher than the therapeutic concentration, caused significant cell death. This finding aligns with the challenge of achieving satisfactory clinical responses when treating malignant tumors with cisplatin alone, highlighting the need for combining cisplatin with other drugs or radiotherapy [160]. De Freitas’s group demonstrated that the sequence of combination therapy yielded different results depending on the photosensitizer used, emphasizing the importance of the treatment order. Low doses of cisplatin acted as sensitizing agents for PG-PDT, while MB-PDT sensitized tumor cells to the action of cisplatin. In conclusion, the combination therapy resulted in enhanced anticancer effects regardless of the treatment protocol. The study suggested that employing low doses of antineoplastic drugs and photosensitizers may maintain treatment efficacy while reducing the likelihood of significant adverse effects [161].

De Freitas et al. conducted a follow-up study using cervical cancer cell lines as a model to investigate the type of cell death induced by PDT and PDT combined with cisplatin, as well as their potential to induce mutations in surviving cells. MB-PDT and PG-PDT induced cell death in the analyzed cervical cancer cell lines, with necrosis and apoptosis, respectively, being the predominant morphological characteristics. MB-PDT predominantly induced necrotic cell death, with approximately two-thirds of dead cells exhibiting necrotic morphology, and one-third showing apoptotic morphology. Cisplatin primarily induced apoptosis, while the combined therapy resulted in varying rates of apoptosis-like or necrosis-like cell death depending on the cell line, with a higher percentage of dead cells compared to monotherapies. These findings support the synergistic effect between PDT and cisplatin and align with the results obtained from cytotoxicity assays. The distinct cell death profiles observed among the cell lines after combined therapy suggest that the cytotoxic effects of each treatment are cell type-dependent. Morphological alterations induced by PG-PDT closely resembled apoptosis. Nevertheless, none of the treatments activated caspase-3, indicating the induction of caspase-independent cell death (CICD) [162]. Although the concept of CICD is relatively recent with limited studies, its characteristics, combining features of both apoptosis and necrosis, suggest a potential role in the development of antitumor immunity [163,164,165]. Notably, some cell lines that undergo caspase-independent cell death exhibit high immunogenicity [165]. Treatment protocols that promote tumor cell death through both apoptosis and necrosis have the potential to elicit antitumor immune responses: necrotic cells can stimulate the necessary inflammatory response to attract immune cells, while apoptotic cells, when phagocytized by professional antigen-presenting cells, can trigger the development of specific antitumor immune responses. De Freitas’s team also observed that MB-PDT, both as a monotherapy and in combination with cisplatin, induced DNA double-strand breaks in all three evaluated cell lines, whereas PG-PDT did not. The mutagenic potential of the evaluated therapies was verified by analyzing treatments for their ability to induce mutations through the micronuclei assay, which assessed the presence of DNA damage. In spite of its genotoxicity, MB-PDT did not exhibit mutagenic effects, as it inhibited the proliferation capacity of surviving cells. The combination of PDT and cisplatin enhances the advantages of each treatment, with low doses of cisplatin administered prior to PG-PDT optimizing the outcome and MB-PDT sensitizing tumor cells to the action of cisplatin. These results highlighted the potential of such combined therapy in reducing the toxicity of antineoplastic drugs. In conclusion, PDT using methylene blue or photogem, whether administered alone or in combination with cisplatin, showed a low likelihood of causing mutations and a potential for stimulating an immune response. The findings provided evidence for the safe and effective use of this therapy in clinical practice for treating cervical cancer [162].

A case report conducted by Ahn focused on two young women who successfully achieved full-term pregnancies and deliveries after undergoing concurrent chemo-photodynamic therapy (CCPDT) for uterine cervical cancer stages 1B1 and 1B2. Case no. 1 involved a 29-year-old single woman diagnosed with squamous cell carcinoma, classified as clinical stage Ib2. After completing the first CCPDT, the lesion significantly improved and returned to an almost normal state. The second CCPDT session further restored the cervix to a perfect normal state. Over the following six years, all follow-up studies, including pap smears, HPV DNA tests, colposcopic biopsies, endocervical curettage, and PET-CT scans, consistently showed normal results. Approximately a year after completing the CCPDT course, she got married and conceived a child through in vitro fertilization (IVF) and embryo transfer (ET). Eventually, she gave birth to a healthy female baby through full-term normal spontaneous delivery at 39 weeks of gestation. Six years after CCPDT, there had been no recurrence of uterine cervical cancer. In case no. 2, a 28-year-old unmarried woman was diagnosed with squamous cell carcinoma, categorized as clinical stage Ib1. The first CCPDT session took place, followed by a second session six weeks later, with the same protocol used as in the previous case. After completing CCPDT, all subsequent follow-up studies showed negative results. Following her marriage, she became spontaneously pregnant and underwent a cesarean operation due to cephalo-pelvic disproportion at 40 weeks and 3 days of gestation, which was 43 months after the CCPDT. There was no evidence of recurrent cancer for a period of six years. According to their study, CCPDT is indicated for the following cases: (1) young women who strongly desire to preserve their uterus and cervix, (2) cervical cancer confined to the cervix (stage 1b or lower), and (3) patients who refuse surgery or radiation therapy. It was noteworthy that this method avoided the need for surgery, complications, recurrences, and adjuvant therapies for uterine cervical cancer. Furthermore, safe full-term deliveries were successfully achieved concurrently. In conclusion, CCPDT represented a new treatment option for uterine cervical cancer that can preserve fertility [155].

In order to address the high toxicity associated with chemotherapy, the development of novel treatment strategies, such as nanosized drug carriers, is crucial. Hoomin Lee et al. introduced biodegradable nanoparticles based on bovine serum albumin (BSA), called folic acid-Ce6/DOX/BSA nanoparticles (FA-Ce6/DOX/BNPs), for targeted chemo-photodynamic combination therapy in cervical cancer. These nanoparticles incorporated both the chemotherapeutic agent DOX and the photosensitizer chlorin e6 (Ce6), enabling synergistic treatment effects. Additionally, FA-Ce6/DOX/BNPs were conjugated with folic acid to specifically target cancer cells that overexpressed folate receptors, such as HeLa cancer cells. To investigate the precise anti-cancer mechanism of FA-Ce6/DOX/BNPs, the researchers evaluated their combined therapeutic effects on HeLa cells [166]. Han Sol Lee et al. also explored the use of hypoxia-alleviating hemoglobin (Hb) nanoclusters (NCs) for sensitizing chemo-PDT in cervical cancer. Hb was conjugated with chlorin e6 and biotinylated polyethylene glycol and adsorbed with DOX, resulting in the self-assembly of protein NCs. These oxygen-carrying NCs, referred to as DOX@HPBC, demonstrated improved colloidal stability in serum compared to native Hb. They also exhibited pH-responsive drug release, which is beneficial for cancer treatment. DOX@HPBC was found to alleviate hypoxia by 64.8% in HeLa cells cultured under hypoxic conditions. It normalized the levels of related biomarkers, HIF-1α and MDR1, and demonstrated enhanced cellular uptake through biotin–receptor interactions. Moreover, in antitumor efficacy tests conducted on HeLa monolayer and spheroid cultures, DOX@HPBC exhibited a 3.8-fold improvement in therapeutic efficacy compared to a physical mixture of DOX and chlorine6. This demonstrated the synergistic effect between chemotherapy and PDT. Overall, the studies mentioned highlight the potential of using nanoparticles for targeted chemo-photodynamic combination therapy in cervical cancer. These approaches offer improved therapeutic efficacy and reduced toxicity, paving the way for the development of new and promising treatment strategies [167].

### 5.2. PDT with Surgery in Cervical Cancer

For women diagnosed with cervical cancer beyond stage IA2 (FIGO stage), the primary treatment options are radical surgery or concurrent chemoradiation, with the latter being a less favored choice. However, neither radical surgery nor radiation therapy preserve fertility. Fortunately, most young cervical cancer patients are diagnosed at an early stage, which has an excellent prognosis after treatment. Radical hysterectomy has shown a 5-year survival rate of 92% in early-stage cervical cancer patients [168]. Consequently, less radical surgery is preferred for young patients with early-stage cervical cancer. In clinical practice, two common types of cervical conization procedures are used: loop electrosurgical excision procedure (LEEP) and cold knife conization (CKC) [169]. CKC can remove a broader range of cervical tissue compared to LEEP, making it suitable for patients with high-grade squamous intraepithelial lesions (HSILs) or those with extensive lesions. However, CKC can lead to the formation of a larger scar tissue area, including a type III transformation zone, which can affect drug absorption [170]. LEEP is a simpler procedure that can be performed on an outpatient basis. However, when performing conization, the main concerns are excising the entire lesion and minimizing the removal of healthy cervical tissue [171]. LEEP can excise lesions within the transformation zone but not those outside it. Additionally, the smoke produced during laser treatment in LEEP may contain active HPV, posing a risk of relapse and resulting in a less than 100% cure rate [172]. Hysterectomy is often considered the definitive treatment for cervical intraepithelial neoplasia (CIN) and invasive cervical cancer. However, it is associated with a known risk factor for the subsequent development of vaginal intraepithelial neoplasia (VaIN), with historical recurrence rates ranging from 0.9% to 6.8% [173,174,175]. The treatment guidelines for VaIN after hysterectomy remain unclear. Different types of treatments are available for VaIN, including drug treatment (trichloroacetic acid, 5-FU, and 5% imiquimod), physical therapy (CO_2_ laser), surgical treatment (partial vaginal resection), and radiotherapy [175,176,177,178]. Each treatment option has its advantages, but there are also limitations and potential complications associated with them. In particular, radiotherapy, while highly effective in treating VAIN and achieving a high cure rate, is not advisable for young patients due to the potential risks it carries, such as vaginal contraction and premature ovarian failure [178]. Recent studies have reported the effectiveness of PDT for the treatment of HPV infection and CIN. PDT selectively targets and kills HPV-infected cells by activating a photosensitizing agent to induce localized photooxidation [179,180,181,182,183]. Combining PDT with LEEP/CKC or hysterectomy has shown potential benefits in reducing recurrence rates and clearing HPV infection. Overall, the current treatment options for cervical cancer and associated conditions have limitations, and there is a need for improved strategies. PDT has emerged as a promising approach, and several studies suggest combining PDT with LEEP/CKC or hysterectomy as potential treatment modalities.

#### 5.2.1. PDT with LEEP in Cervical Cancer

Choi et al. performed a retrospective study to evaluate the effectiveness of PDT as a conservative treatment option for fertility preservation in early-stage cervical cancer patients. The study analyzed the medical records of 21 patients diagnosed with cervical cancer. In every case, patients underwent LEEP or conization prior to PDT. For patients with stage IB1 or above, only those who were confirmed to be free of malignancy in frozen section analysis by pelvic lymph node dissection received PDT. PDT was performed after neoadjuvant chemotherapy based on the response to the chemotherapy treatment. In terms of stage distribution, 10 patients (47.6%) were classified as stage IA1, one patient (4.7%) as stage IA2, nine patients (42.9%) as stage IB1, and one patient (4.7%) as stage IIA1. Histologically, 17 patients (80.9%) had squamous cell type, two patients (9.5%) had adenocarcinoma type, and the remaining two patients (9.5%) had glassy cell type. Among the 10 patients in stage IB1 or above, five patients had lesions with a diameter of 2 cm or larger. The five patients who underwent neoadjuvant chemotherapy showed a reduction in the size of the lesion by more than 50% based on imaging studies and colposcopic examination. During the follow-up period, there was one recurrence (4.7%) and no deaths observed over a median duration of 52.6 months (ranging from 6 to 114 months). Thirteen patients attempted to conceive, and among them, 10 patients achieved a total of 11 pregnancies. Out of these pregnancies, there were two cases of missed abortions, one ectopic pregnancy, seven cases of live births, one stillbirth, and one neonatal death (from a set of twins). Regarding gestational weeks, there were five cases of full-term gestation (more than 37 weeks), no cases of preterm birth between 34 and 37 weeks, and three cases of preterm birth between 25 and 34 weeks. The study was significant as it represented a pilot study on the application of PDT with LEEP or conization for early-stage cervical cancer patients with the goal of fertility preservation [184].

Kim et al., who are colleagues of Choi’s team, conducted a separate study to investigate the long-term outcomes of PDT in cases of incomplete excision of CIN3 (cervical intraepithelial neoplasia grade 3). CIN is a premalignant lesion associated with persistent high-risk human papillomavirus (HPV) infection and is categorized into CIN1 to CIN3 based on the degree of dysplasia [185]. When test results indicate CIN2+ (CIN2 or higher), a colposcopic biopsy is recommended. Standard treatments for non-pregnant patients with histologically confirmed CIN3+ involve diagnostic excisional procedures such as CKC or LEEP. Although these procedures typically result in complete cure, recurrence rates of 6.0–16.5% within 5 years after treatment have been reported [186,187]. The aim of the study was to investigate the long-term outcomes of PDT in patients with positive resection margins following conization or LEEP for CIN3+. The researchers retrospectively reviewed the medical records of 73 patients who underwent PDT for CIN. Ultimately, 34 patients met the inclusion criteria for the study. The majority of patients in the study exhibited clinicopathologic conditions associated with an increased risk of residual disease or recurrence after conization or LEEP. Approximately 76.5% (26/34) of the patients had involvement of the endocervical resection margin, and 76.5% (26/34) had glandular involvement. The complete response (CR) rate after 1 year was 97.1%. With the exception of one case of persistent disease, no recurrence or new disease was observed during the median follow-up period of 84 months. Initial HPV testing yielded positive results in 32 patients (94.1%), and all cases of HPV infection were of the high-risk type. Among the patients, 27 had a single HPV infection, while multiple HPV infections (two or more HPV types) were detected in the remaining patients. The HPV eradication rate following PDT after 6 months was 96.9% (31/32). At 1 year after PDT, five patients still had positive HPV test results, with three of them showing the same HPV type as before treatment. However, these infections were transient, as subsequent HPV testing showed negative results. Throughout the long-term follow-up period, all patients, except those with transient infections, consistently showed negative HPV test results. The study demonstrated a higher eradication rate compared to that of conization/LEEP or PDT alone, suggesting a synergistic effect of conization/LEEP and PDT on HPV eradication. Throughout the follow-up period, a total of 14 pregnancies were detected in 10 patients who underwent PDT. In conclusion, this study demonstrated that PDT reduced the risk of recurrence or residual lesions following incomplete excision of CIN3+ without severe adverse outcomes. PDT could be considered as an alternative treatment option for patients with positive resection margins who wish to preserve their uterus [188].

Cai et al. conducted a study using ALA-PDT as part of the treatment plans for six patients with persistent CIN following LEEP, aiming to enhance the overall therapeutic effectiveness. ALA-PDT, which involves the use of 5-aminolevulinic acid, is a targeted therapy that focuses on HPV-infected sites. Previous reports have shown successful treatment of high-grade squamous intraepithelial lesions with a combination of LEEP conization and ALA-PDT. The therapeutic effects of the combined treatment on CIN were evaluated using a double screening system that included HPV DNA detection and liquid-based cervical cytology (LCT). The results were reported on a scale of 1–3: (1) no intraepithelial lesions or neoplasia; (2) atypical squamous cells of undetermined significance (ASCUS); (3) low-grade squamous intraepithelial lesion (LSIL). In the study, six patients with persistent cervical abnormalities following LEEP were treated with four to seven sessions of ALA-PDT. All six patients responded well to ALA-PDT, showing no abnormalities on LCT at 6–7 months post-treatment. Five of the six patients also tested negative for HPV, but a longer follow-up period is necessary to confirm HPV eradication. The study recommended ALA-PDT as an adjunct treatment to LEEP for high-grade CIN patients, with an approximate 3-month interval between LEEP and starting ALA-PDT. In conclusion, ALA-PDT has shown great potential as an adjunct treatment for high-grade CIN when combined with LEEP for lesion removal. The interval of approximately 3 months between LEEP and ALA-PDT had a critical impact on the patients’ recovery process. Emphasizing daily care and promoting a healthy lifestyle before and after the therapy was crucial for supporting recovery. Regular clinical examinations were necessary to detect any signs of recurrence, even if HPV DNA and LCT test results were satisfactory. Regular reviews enabled timely intervention based on the patient’s condition. Overall, ALA-PDT holds promise as a supplementary treatment option for patients with persistent CIN [172].

Wang et al. conducted a study to assess the effectiveness and safety of PDT in women with high-risk human papillomavirus (hr-HPV) persistent infection after cervical conization. The study included 76 women who had undergone cervical conization, either by LEEP or CKC. The main outcome measure was the HPV negative conversion rate after PDT. Complete remission was defined as the total clearance of HPV without new genotype infection at the end of follow-up. Partial remission referred to patients with multi-type HPV infection where HPV was not completely cleared. Non-remission indicated no change in HPV genotypes. Progression was defined as the absence of negative HPV results and new infection or the emergence of lesions of ≥ CIN1 grade. Among them, 27 cases were positive for HPV16/18 and 49 cases for other hr-HPVs. During the 12-month follow-up period, one patient aged 60 with persistent HPV16 infection underwent hysterectomy due to progression to cervical HSIL involving glands, and seven patients were lost to follow-up (four patients showed no remission and three showed partial remission at the 6-month follow-up). At 6 months after PDT, the overall HPV clearance rate was 59.21% (45/76). The HPV negative conversion rates were 68.52% (37/54) for patients ages ≤50 years and 36.36% (8/22) for patients ages >50 years. However, there was no significant difference in HPV clearance rate between the HPV16/18 infection group and the other hr-HPV infection group. The overall HPV clearance rate was 88.24% (60/68). The negative conversion rates for HPV16/18 and other hr-HPV infection groups were 76.00% (19/25) and 95.35% (41/43), respectively. The HPV negative conversion rate did not correlate with patient age. Mild adverse reactions after PDT were observed, mainly increased vaginal secretions or burning/tingling. In conclusion, PDT demonstrated effectiveness in treating patients with hr-HPV persistent infection after cervical conization, promoting negative HPV conversion, and preventing recurrence or progression of cervical intraepithelial neoplasia following LEEP/CKC [170].

#### 5.2.2. PDT with Hysterectomy in Cervical Cancer

Huang et al. conducted a study to evaluate the efficacy PDT for treating persistent vaginal high-risk human papillomavirus infection in post-hysterectomy patients with or without vaginal intraepithelial neoplasia (VaIN). The study included patients who had a history of hysterectomy for cervical lesions or other benign lesions of the uterus and were followed up after the operation. These patients were found to have persistent vaginal stump hr-HPV infection for more than 12 months, with or without pathologically diagnosed VaIN1 during the follow-up period. A total of 38 patients with persistent vaginal stump hr-HPV infection with or without histological VaIN1 were recruited for the study. All patients with VaIN had hysterectomy due to CIN/CC. Out of the 20 patients in the PDT group, four (20%) had HPV16/18-related infections, while 16 (80%) had other hr-HPV infections. In comparison, the control group consisted of 18 patients who did not receive any treatment. The hr-HPV remission rates in the PDT group were 40% (8/20) and 66.67% (12/18) at the 4–6-month and 12-month follow-up, respectively. These rates were significantly higher than those in the control group (11.11% (2/18) and 6.23% (1/16), respectively). In the PDT group, VaIN1 regression was observed in all seven cases (100%) at the 4–6-month follow-up, but recurrence occurred in two cases (28.6%) at the 12-month follow-up due to persistent hr-HPV infection. No persistence or progression of VaIN1 was noted. In the control group, spontaneous regression of VaIN1 was observed in three out of six cases (50%) at the 4–6-month follow-up, and one patient progressed to VaIN2. At the 12-month follow-up, one patient experienced recurrence, resulting in regression rates of 40% (2/5), persistence rates of 40% (2/5), and recurrence rates of 20% (1/5). Adverse reactions after PDT treatment were mild. Most patients (18/20, 90%) reported a sensation of local vaginal burning or tingling, which was relieved by oral analgesics. Five patients (25%) experienced increased vaginal secretion, which resolved spontaneously after treatment. In conclusion, 5-ALA-PDT was proven to be a non-invasive and effective treatment for patients with persistent hr-HPV infection after hysterectomy. It did not cause serious adverse reactions and preserved the integrity of the reproductive tract structure and function. Therefore, it could be a suitable option for high-risk patients with persistent HPV infection and a history of hysterectomy due to cervical lesions [189].

Zhao et al. conducted a study to investigate the impact of ALA-PDT on high-grade vaginal intraepithelial lesions (HG VAIN) in patients who had undergone hysterectomy and were infected with high-risk human papillomavirus (hr-HPV). The study also aimed to assess the clinical effectiveness and safety of ALA-PDT in patients with HG VAIN after hysterectomy and HPV infection. The clinical data of 23 patients with HG VAIN following hysterectomy were collected. These patients had undergone hysterectomy for reasons related to non-cervical or cervical factors. Persistent HPV infection was observed before and after the surgery, and during the follow-up, they were diagnosed with high-grade vaginal intraepithelial lesions (VAIN II-III) after hysterectomy. After hysterectomy, 43.5% of patients (10 cases) were diagnosed with VAIN II, 34.8% (8 cases) had VAIN III, and 21.7% (5 cases) had VAIN II-III. Hysterectomy due to cervical factors accounted for 60.9% (14 cases), while hysterectomy due to non-cervical factors accounted for 39.1% (9 cases). All patients had hr-HPV infection, including 15 cases of HPV16 infection (65.2%), 1 case of HPV16/18 infection (0.04%), 3 cases of HPV18 infection (13.0%), and 4 cases of other types of hr-HPV infection (17.4%). The therapeutic effect, adverse reactions, recurrence rate, and HPV clearance rate were assessed in the study. After 3 months of PDT, 21 patients were cured, resulting in a cure rate of 91.3% (21/23). Two patients (8.7%) had residual lesions but with improved conditions compared to before treatment. The overall hr-HPV clearance rate was 56.5% at 3 months, 65.2% at 6 months, 69.5% at 9 months, and 74% at 12 months. No significant adverse reactions were observed during or after treatment, and no recurrence occurred during the entire follow-up period. ALA-PDT, compared to other therapies, offered a novel non-invasive approach for the management of HG VAIN following hysterectomy. The study demonstrated that ALA-PDT effectively cleared hr-HPV, offering potential for preventing the recurrence and progression of vaginal lesions associated with persistent hr-HPV infection [190].

**Table 4:** Combined therapies with PDT in cervical cancer.

Combined Therapy		Photosensitizer	Dose	Light Source	Study Type	Ref.
Chemotherapy	cisplatin (0.1~20 mg/L)	5-ALA	0.1~4 mM/L	635 nm (5 J/cm^2^)	in vitro	[154]
	carboplatin (0–1000 µM)	Photofrin^®^	20 µM	630 nm (0–3300 mJ/1.32 cm^2^)	in vitro	[158]
	cisplatin (1.3–166 µM)	Photogem^®^, Methylene blue	5 mg/mL 10 mg/mL	[PG-PDT] 630 nm (1.39 or 2.76 J/cm^2^) [MB-PDT] 660 nm (1.29, 2.56, 5.11 or 12.9 J/cm^2^)	in vitro	[161]
	cisplatin (1.3 μM)	Photogem^®^, Methylene blue	0.5 μM 19.5 μM	[PG-PDT] 630 nm (2.76 J/cm^2^) [MB-PDT] 660 nm (5.11 J/cm^2^)	in vitro	[162]
	carboplatin (75 mg/m^2^)	Photogem^®^, Photofrin^®^	2.5 mg/kg, 2 mg/kg	(240 J/cm^2^, exocervix) (250 J/cm, endocervix)	clinical	[155]
Surgery	LEEP, conization	Photogem^®^	2 mg/kg	630 nm (150 J/cm^2^, cervix) (200 J/cm^2^, endocervical canal)	clinical	[184]
	LEEP, conization	Photogem^®^	2 mg/kg	630 nm	clinical	[188]
	LEEP	5-ALA	20% solution	635 nm (80 mw/cm^2^)	clinical	[172]
	LEEP, conization	5-ALA	20% solution	632.8 nm (100 J/cm^2^)	clinical	[170]
	hysterectomy	5-ALA	20% solution	635 nm (100 J/cm^2^)	clinical	[189]
	hysterectomy	5-ALA	20% solution	635 nm (80 J/cm^2^)	clinical	[190]

## 6. Conclusions

In conclusion, this review underscores the promising role of PDT in addressing the limitations of traditional cancer treatments across various malignancies, including lung cancer, breast cancer, cholangiocarcinoma, and cervical cancer. The exploration of PDT’s synergistic applications with chemotherapy, surgery, and radiotherapy highlights its potential to enhance treatment outcomes in diverse clinical scenarios. The acknowledgement of the constraints associated with conventional cancer therapies emphasizes the growing significance of combination approaches involving PDT. The comprehensive analysis of different cancer types and their combination therapies provides valuable insights into the evolving landscape of cancer treatment strategies. As research and clinical efforts continue to advance, the integration of PDT with traditional modalities emerges as a promising avenue for improving therapeutic efficacy and overcoming challenges in cancer care. This review contributes to the existing body of knowledge, offering a foundation for further research and supporting clinicians in their pursuit of innovative and effective cancer treatment approaches.

Recent research has introduced PDT-based combination cancer therapies that utilize various advanced drug delivery systems, such as cold-responsive upconversion nanoparticles [191], aggregation-induced emission photosensitizer-engineered nanoplatforms [192], platelet membrane-based biochemotactic-targeting nanomedicines [193], and reduction-responsive worm-like nanoplatforma [194]. These innovative naoplatform-based approaches to PDT and combination cancer therapies hold great potential to accelerate advancements in cancer treatment.

However, the combination of PDT with traditional treatments may increase financial burdens on patients and necessitate more frequent hospital visits. To address these challenges, future research should focus on strategies to streamline PDT applications with other therapies, minimizing patient costs and treatment intervals. Overall, this review contributes to the body of knowledge on PDT, offering a foundation for future research and supporting clinicians in their pursuit of innovative and effective cancer treatment strategies.

## Abbreviations

5-ALA	5-aminolevulinic acid
ASCUS	atypical squamous cells of undetermined significance
BCS	breast-conserving surgery
BDC	bile duct carcinoma
BSA	bovine serum albumin
BW	body weight
CCPDT	concurrent chemo-photodynamic therapy
CDDP	cis-diamminedichloroplatinum
CICD	caspase-independent cell death
CIN	cervical intraepithelial neoplasia
CKC	cold knife conization
CPBN	capecitabine
CR	complete response
Cu-Cy	copper-cysteamine
DOX	doxorubicin
EPR	enhanced permeability and retention
ERCP	endoscopic retrograde cholangiopancreatography
FDA	Food and Drug Administration
FIGO	International Federation of Gynecology and Obstetrics
GaPcCl	gallium phthalocyanine chloride
HDR	high dose rate
HDRILBT	high dose rate intraluminal brachytherapy
HG VAIN	high-grade vaginal intraepithelial lesions
HpD	hematoporphyrin derivative
hr-HPV	high-risk human papillomavirus
HSIL	high-grade squamous intraepithelial lesions
ICG	indocyanine green
LCT	liquid-based cervical cytology
LDH	lactate dehydrogenase
LEEP	loop electrosurgical excision procedure
LSIL	low-grade squamous intraepithelial lesion
MB	methylene blue
MB-DOX	doxorubicin-methylene blue
MT	mitoxantrone
nanoMB	biocompatible nanocomplex formulation of methylene blue
NCC	nonresectable cholangiocarcinoma
NOZ	bile duct carcinoma cell line
NSCLC	non-small cell lung carcinoma
PCNA LI	proliferating cell nuclear antigen labeling index
PDT	photodynamic therapy
PDX	patient-derived xenograft
PG	photogem
pHLIP	pH-low insertion peptide
PpIX	protoporphyrin IX
PS	photosensitizer
PTCS	percutaneous transhepatic choledochoscopy
PVIA	percentage of the VEGF-immunopositive area
QOL	quality of life
RCI	Reid colposcopic index
ROS	reactive oxygen species
S-1	oral fluoropyrimidine prodrug that contains tegafur, 5-chloro-2,4-dihydroxypyridine (CDHP), and potassium oxonate (Oxo)
SCLC	small cell lung carcinoma
TBARS	thiobarbituric acid reactive substance
TCT	thinprep cytologic test
TPS	talaporfin sodium
UHC	unresectable hilar cholangiocarcinoma
X-PDT	X-ray induced photodynamic therapy
ZnPcS-PDT	sulfonated zinc phthalocyanine-mediated photodynamic therapy
